# The C/D box small nucleolar RNA SNORD52 regulated by Upf1 facilitates Hepatocarcinogenesis by stabilizing CDK1

**DOI:** 10.7150/thno.47677

**Published:** 2020-07-23

**Authors:** Cuicui Li, Long Wu, Pengpeng Liu, Kun Li, Zhonglin Zhang, Yueming He, Quanyan Liu, Ping Jiang, Zhiyong Yang, Zhisu Liu, Yufeng Yuan, Lei Chang

**Affiliations:** 1 Department of Integrated Internal Medicine and Geriatrics, Zhongnan Hospital of Wuhan University, Wuhan 430071, P.R. China.; 2 Department of Hepatobiliary and Pancreatic Surgery, Zhongnan Hospital of Wuhan University, Wuhan 430071, P.R. China.

**Keywords:** Hepatocellular carcinoma, Upf1, SNORD52, Hepatocarcinogenesis

## Abstract

**Rationale:** Understanding the roles of small nucleolar RNAs (snoRNAs) in hepatocarcinogenesis will provide new avenues to identify diagnostic and therapeutic targets for hepatocellular carcinoma (HCC). Our previous research confirmed the tumor-suppressive effect of Up-frameshift 1 (Upf1) in HCC. Herein, we examined the expression profiles of snoRNAs regulated by Upf1 in hepatoma cells.

**Methods:** We examined the expression profiles of snoRNAs regulated by Upf1 in hepatoma cells using RNA-sequencing analysis and then investigated the expression and significance of SNORD52 in HCC tissue and different cell lines. The protumorigenic effects of SNORD52 on HCC cells were confirmed both *in vitro* and *in vivo* by gain-of-function and loss-of-function assays. RNA pull-down assays and mass spectrometry were used to identify the RNA-binding protein that binds to SNORD52.

**Results:** Many snoRNAs were identified; one of which, the human C/D box small nucleolar RNA SNORD52, was upregulated in HCC tissues and negatively correlated with Upf1 expression, and patients with higher SNORD52 expression had a poor clinical prognosis. SNORD52 promoted HCC tumorigenesis both *in vitro* and *in vivo*. Mechanistically, KEGG analysis showed that SNORD52 upregulated a series of cell cycle genes in HCC cells. We further confirmed that SNORD52 upregulated CDK1 by enhancing the stability of CDK1 proteins and that the function of SNORD52 depends on the presence of CDK1.

**Conclusion:** Overall, the present study indicates that SNORD52 could be a potential biomarker for HCC. Targeting the Upf1/SNORD52/CDK1 pathway might have therapeutic potential for HCC.

## Introduction

Hepatocellular carcinoma (HCC), which accounts for more than 90% of liver cancers, is one of the most common malignant tumors, and its incidence is on the rise worldwide 1. It is the second leading cause of cancer-related deaths in the Asia-Pacific area, especially in China, and poses a serious threat to the lives and health of people all over the world 2. In recent years, despite the development of clinical therapies and molecular-targeted drugs, the morbidity and mortality of HCC have remained high. Once HCC is diagnosed, most patients are in intermediate and advanced stages and have already missed the optimal treatment time. The poor prognosis of HCC is closely related to the biological behaviors of tumor cells, such as rapid growth and high metastasis potential, but the molecular mechanism of HCC has not been clarified. Therefore, exploring the molecular mechanism of HCC tumorigenesis has very important clinical value and significance for the diagnosis and treatment of HCC.

Small nucleolar RNAs (snoRNAs) are a subgroup of noncoding RNAs (ncRNAs) with lengths of 60-300 nucleotides, predominately found in the nucleolus. According to their conserved sequences and structural elements, snoRNAs can be divided into three categories: C/D box snoRNA, H/ACA box snoRNA and ribonuclease 7-2/MRP. Among them, C/D and H/ACA are the main types of known snoRNAs 3, 4. Regarding the function of snoRNAs, they have long been known to modify and make rRNA mature and stable, and this posttranscriptional modification is important for ribosomal biogenesis 5. Recent studies have shown that snoRNAs can indirectly regulate gene expression by processing to generate small noncoding RNAs, such as miRNAs and piwi-interacting RNAs (piRNAs), and they play an important role in carcinogenesis 6. snoRNAs can be used as tumor markers and can not only affect the occurrence and development of tumors by affecting the expression of its host genes but also affect the prognosis of tumor patients. In addition, snoRNAs can also be used as new targets for the treatment of cancer patients. SNORA42 can be used as a biomarker to predict the prognosis of colorectal cancer 7. In non-small cell lung cancer (NSCLC), the inhibition of SNORD78 expression could induce cell cycle arrest in the G0/G1 phase, thereby inhibiting the proliferation of NSCLC cells 8. SNORA23 regulated the metastasis of pancreatic cancer cells by upregulating SYNE2 in pancreatic ductal adenocarcinoma (PDAC) 9. The human C/D box small nucleolar RNA 52 (SNORD52) (Accession: LN848124.1) located on chromosome 6p21.33, with 64 nucleotides in length 10. SNORD52 is encoded by the intron of snoRNA host gene 32 (SNHG32). Up to now, the function of SNORD52 is largely unknown.

Nonsense-mediated mRNA decay (NMD) is a common mRNA surveillance pathway in eukaryotes and degrades mRNA containing a premature termination codon (PTC), preventing the synthesis of potentially toxic truncated proteins with no biological activity 11. Gene expression profiling studies have shown that during the development of the body, the depletion of NMD factors can lead to the dysregulation of 3%-15% of normal transcripts 12. As the core transacting factor of NMD, Up-frameshift 1 (Upf1), an ATP-dependent RNA helicase with a size of 123~124 kDa, is located in the cytoplasm. Upf1 was originally discovered in Saccharomyces cerevisiae; it hydrolyzes ATP and then expands RNA from the 5' to 3' direction 13. Studies have found that the exon junction complex (EJC) is formed 20 nt upstream of the exon-exon junction (EEJ), which is generated after mRNA splicing. When translation is terminated early, Upf1 combines with the translation release factors eRFl and eRF3 and then triggers NMD 14. Recent studies have shown that Upf1 is dysregulated in various tumors and plays an important role in carcinogenesis 15-17. Liu et al. found that Upf1 was downregulated in pancreatic adenosquamous carcinoma (ASC) and that the Upf1 gene was commonly mutated in ASC. The discovery of Upf1 gene mutations in ASC represents the first known example of NMD gene mutations in human tumors 18. Our previous study indicated that Upf1 is a potential tumor suppressor gene that regulates hepatocarcinogenesis by targeting Smad7. Furthermore, Upf1 was closely related to the prognosis of patients with HCC 19. This is the first report on the role of Upf1 in hepatocarcinogenesis. To date, no studies have assessed the particular relationships between Upf1 and snoRNAs in human HCC, which prompted our interest in investigating the biological roles of snoRNAs regulated by Upf1 in hepatocarcinogenesis.

In the present study, we examined the expression profiles of snoRNAs in HCC cells by performing high-throughput RNA sequencing (RNA-seq). We identified C/D box small nucleolar RNA 52 (SNORD52) as an Upf1-regulated snoRNA. SNORD52 was upregulated in HCC tissues and was closely associated with poor prognosis in patients with HCC. SNORD52 exhibited an oncogenic effect in HCC tumorigenesis both *in vitro* and *in vivo*. Mechanistically, SNORD52 combined with CDK1 and increased its protein level by enhancing its stability in HCC. Based on these findings, the Upf1-SNORD52-CDK1 pathway may provide a promising strategy for the targeted therapy of HCC.

## Materials and Methods

### Ethical application

The protocols used in this study complied with the ethical guidelines of the 1975 Declaration of Helsinki and were approved by the Medical Ethics Committee of Zhongnan Hospital of Wuhan University (Wuhan, China) (Scientific Ethical Approval No. 2017052).

### Human HCC tissue samples

A retrospective cohort of 80 HCC patients who underwent routine surgery at Zhongnan Hospital of Wuhan University (Wuhan, China) was included in this study. Ethical approval was obtained from the Medical Ethics Committee of Zhongnan Hospital of Wuhan University, and written informed consent was obtained from each patient. The pathological diagnosis of HCC was performed according to World Health Organization (WHO) standards. The clinical data of HCC patients were acquired from the electronic medical records of the Hepatobiliary and Pancreatic Surgery Department of Zhongnan Hospital. The inclusion criteria were as follows: primary diagnosis of HCC from 2004 to 2014 (at least 5 years of follow-up); no previous diagnosis of other cancers; no other underlying diseases within one month before surgery; and no neoadjuvant therapy such as chemotherapy or radiotherapy before surgery. Specimens were stored at -80 °C in an RNA stabilization solution immediately for further assays. Overall survival was defined as the interval between surgery and death or the last follow-up visit. Disease-free survival was defined as the interval between surgery and the date of clinical relapse.

### Cell culture and reagents

The human hepatoma cell lines used in this study, including Huh7, HepG2, Hep3B, SK-Hep1, HCCLM9, and HCCLM3, and the immortalized human hepatic cell line HL-7702 were purchased from the Cell Bank of Type Culture Collection (CBTCC, Shanghai, China). All cell lines were tested for mycoplasma, DNA fingerprinting, isozymes and cell viability by a third-party biological service organization (GeneCreate Biology Co., Ltd., Wuhan, China). The cells were cultured in a 37 °C humidified incubator with 5% CO_2_ in RPMI-1640 medium (Invitrogen, USA) supplemented with 10% fetal bovine serum (Gibco, USA). Cycloheximide (CHX) was purchased from Sigma (St. Louis, MO). MG-132 reagent was purchased from Merck Chemicals Ltd. (Darmstadt, Germany). Conditions for treatment without specific notification were CHX (0.5 μg/μL), and MG132 (5 μM).

### RNA isolation and quantitative real-time PCR

TRIzol reagent (Invitrogen, USA) was used to extract the total RNA from tissues and cells according to the manufacturer's protocol. PrimeScript™ RT Reagent (Takara, Japan) was used to reverse transcribe cDNA. SYBR Green mix (Toyobo, Japan) was used for quantitative real-time PCR, which was performed using the CFX Connect Real-Time PCR Detection System (Bio-Rad, USA). GAPDH or U6 was used as an internal control, and three independent experiments were performed for each sample. The primers used in this study are listed in **Table S1**.

### RNA interference, plasmid construction and cell transfections

The siRNA and antisense oligonucleotide (ASO) used in this study to knockdown Upf1, CDK1 and SNORD52 expression were designed and synthesized by RiboBio (Guangzhou, China). First-strand SNORD52 cDNA was synthesized by using the SuperScript III First-Strand Synthesis System (Invitrogen, USA) with gene-specific primers. The full-length SNORD52 sequence was then cloned into the expression vector pCMV (Invitrogen, USA) for SNORD52 overexpression. siRNA and ASO transfection were performed using riboFECT™ CP Reagent (RiboBio, Guangzhou, China), and plasmids were transfected with Lipofectamine 2000 (Life Technologies, USA) according to the manufacturer's protocol. ASOs used *in vivo* with the same sequences were chemically modified by RiboBio (Guangzhou, China). The modifications were described previously 20, 21.

### RNA pull-down assay and mass spectrometry

SNORD52 and its antisense RNA were synthesized *in vitro* by RiboBio (Guangzhou, China) and biotin-labeled by using the Biotin RNA Labeling Kit (Roche, USA). Then, the RNeasy Mini Kit (Qiagen, Germany) was used to purify the product. The Pierce Magnetic RNA-Protein Pull-Down Kit (Thermo Scientific, USA) was used in this study for the RNA pull-down assay. In short, HCC cell lysates and streptavidin magnetic beads were incubated with biotin-labeled SNORD52 RNA and its anti-sense RNA and then washed. Subsequently, the proteins bound to the streptavidin magnetic beads were identified and separated by SDS-PAGE. Finally, the gel was stained by silver, and the specific fragments of SNORD52 sense sequence which had significant difference compare with antisense sequence were excised for mass spectrometry (Ekspert^TM^ nanoLC, Shanghai, China) or western blotting.

### RNA immunoprecipitation (RIP) assay

The RIP assay was performed using the Magna RIP™ RNA-Binding Protein Immunoprecipitation Kit (Millipore, USA) according to the manufacturer's instructions. In brief, HCC cells were harvested and treated with RIP lysis buffer. Negative control IgG and human anti-CDK1 antibodies were immunoprecipitated with A/G magnetic beads. The magnetic bead-bound complexes were immobilized with a magnet, and the unbound complexes were washed off. Finally, total RNA was extracted and subjected to quantitative real-time PCR. The quality of RNA was assessed by a Nanodrop^TM^ 2000 spectrophotometer (Thermo Scientific, USA). The enrichment level of RNA was normalized to that of the input and compared to that of IgG. Agarose gel electrophoresis was also conducted to observe the fragments of cDNA.

### Other experimental procedures for *in vitro* and *in vivo* assays are described in the Supplementary Information

### Statistical analysis

All data included in this study are presented as the mean ± standard deviation (S.D.) from at least three independent experiments. Data analyses were performed using Prism 8.3.0 (GraphPad Software, USA). Student's t-test, the Wilcoxon signed-rank test, Fisher's exact test, the χ^2^ test and the Mann-Whitney *U* test were used for comparisons between groups, as appropriate. The Kaplan-Meier method was used to evaluate survival. Linearity was evaluated by Pearson's correlation analysis. Multivariate Cox regression analysis was utilized to predict the independent prognostic factors. *P* <0.05 indicated that the difference was statistically significant.

## Results

### Identification of SNORD52 as an Upf1-regulated snoRNA

**Figure 1:**
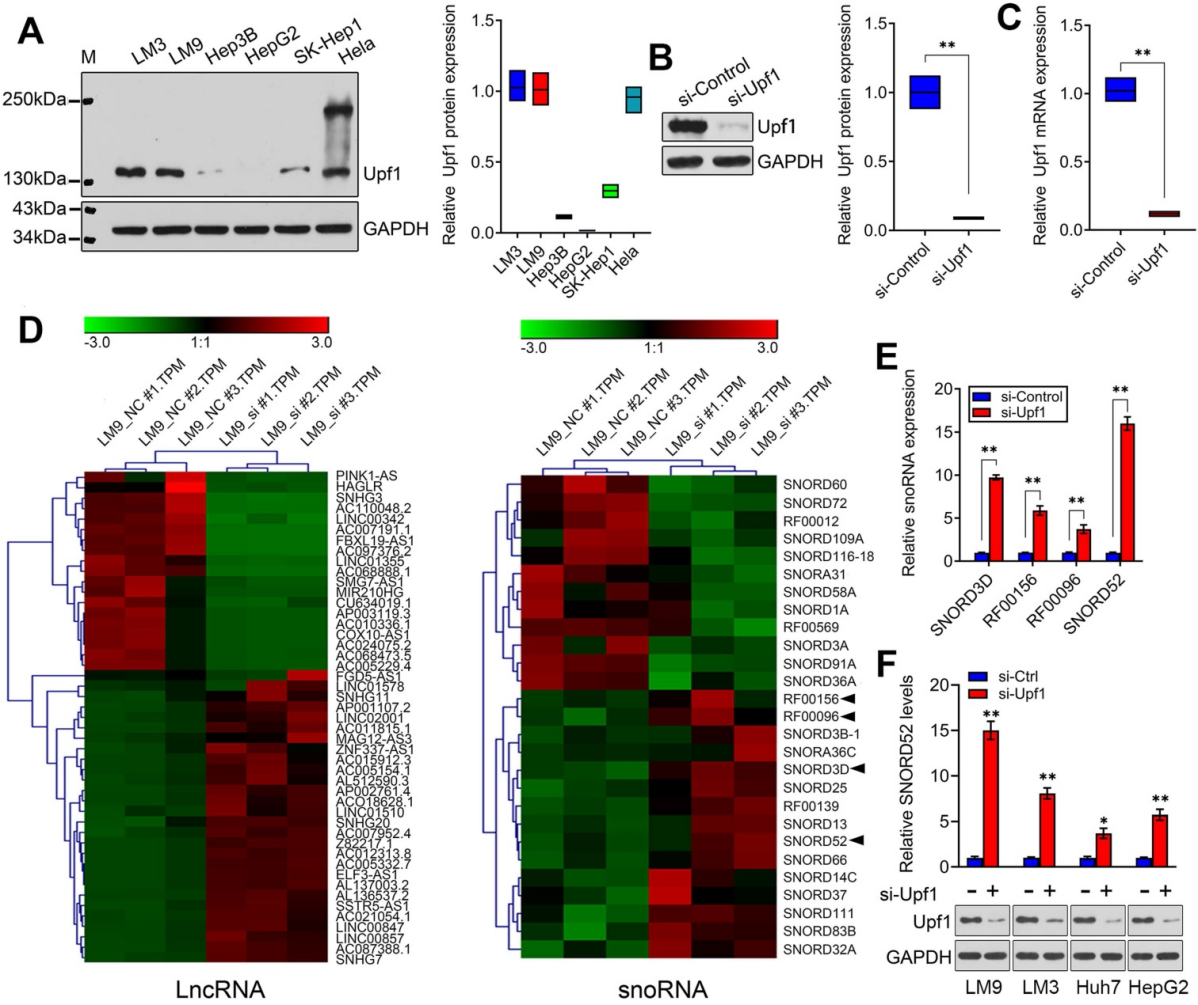
** Identification of SNORD52 as an Upf1-regulated snoRNA. (A)** Western blotting showed Upf1 protein levels in hepatoma cells and HeLa cells. **(B-C)** Upf1 was knocked down using siRNA, and western blotting and quantitative RT-PCR showed the Upf1 protein and mRNA expression levels in HCCLM9 cells. Student's t-test, *p<0.05, **p<0.01. **(D)** Three samples were analyzed for the Upf1-siRNA group and the control group. The clustering tree for lncRNAs and snoRNAs is shown in the right panel. The expression values are represented in shades of red and green, indicating expression above and below, respectively, the median expression values across all of the samples (log scale of lncRNA: from -3.0 to 3.0; log scale of snoRNA: from -3.0 to 3.0). The arrow indicates the candidate snoRNAs for further research. **(E)** The expression levels of SNORD3D, RF00156, RF00096 and SNORD52 in HCCLM9 cells were detected by quantitative RT-PCR when Upf1 was knocked down. The data are shown as the mean±S.D. based on at least three independent experiments. *p<0.05, **p<0.01. **(F)** Western blotting and quantitative RT-PCR showed the Upf1 protein and SNORD52 expression levels in the indicated HCC cells when Upf1 was knocked down. *p<0.05, **p<0.01.

Although some snoRNAs have been found in HCC 6, the genome-wide screening of Upf1-regulated snoRNAs has not been reported, and the specific regulatory mechanisms involving snoRNAs during HCC development are not well understood. Given the close connection between Upf1 and various noncoding RNAs, we speculate that Upf1 may also regulate snoRNAs, as snoRNAs are related to carcinogenesis. To confirm our hypothesis, first, the expression level of Upf1 in HCC cell lines and HeLa cells was detected using western blotting (**Figure 1A**). Due to the high expression level of Upf1 in HCCLM9, we chose HCCLM9 as the main cell line for further study. We knocked down the expression level of Upf1 in HCCLM9 cells using RNAi (**Figure 1B,C**). By using high-throughput RNA sequencing, a differential expression profile was obtained for each group by comparing the microarray signal with that obtained from HCCLM9 cells. Hierarchical clustering showed the dysregulation of large amounts of noncoding RNAs, and a total of 47 lncRNAs and 27 snoRNAs were differentially expressed (fold change >2.0) in HCCLM9 cells between the si-Upf1 group and si-Control group (**Figure 1D**), (**Table S3 and Table S4**). The top 4 of the 27 dysregulated snoRNAs were SNORD3D, RF00156, RF00096 and SNORD52, so we chose the above 4 snoRNAs for further study. To identify the oncogenic snoRNAs regulated by Upf1 that significantly affect HCC development, we knocked down the expression level of Upf1 in HCCLM9 cells. Quantitative RT-PCR analysis showed that SNORD3D, RF00156, RF00096 and SNORD52 were upregulated when Upf1 was silenced, and SNORD52 had the most obvious change in expression level (**Figure 1E**). In addition, the expression level of SNORD52 was significantly upregulated when Upf1 was knocked down in HCCLM9, HCCLM3, Huh7 and HepG2 cells (**Figure 1F**). Collectively, these data suggest that SNORD52 may be one of the Upf1-repressed targets. The additional RNA-seq results are presented in **Figure S1 and Figure S2**.

### SNORD52 was significantly upregulated in HCC tissues and negatively correlated with the expression of Upf1, and high SNORD52 expression is associated with poor prognosis in HCC

**Figure 2:**
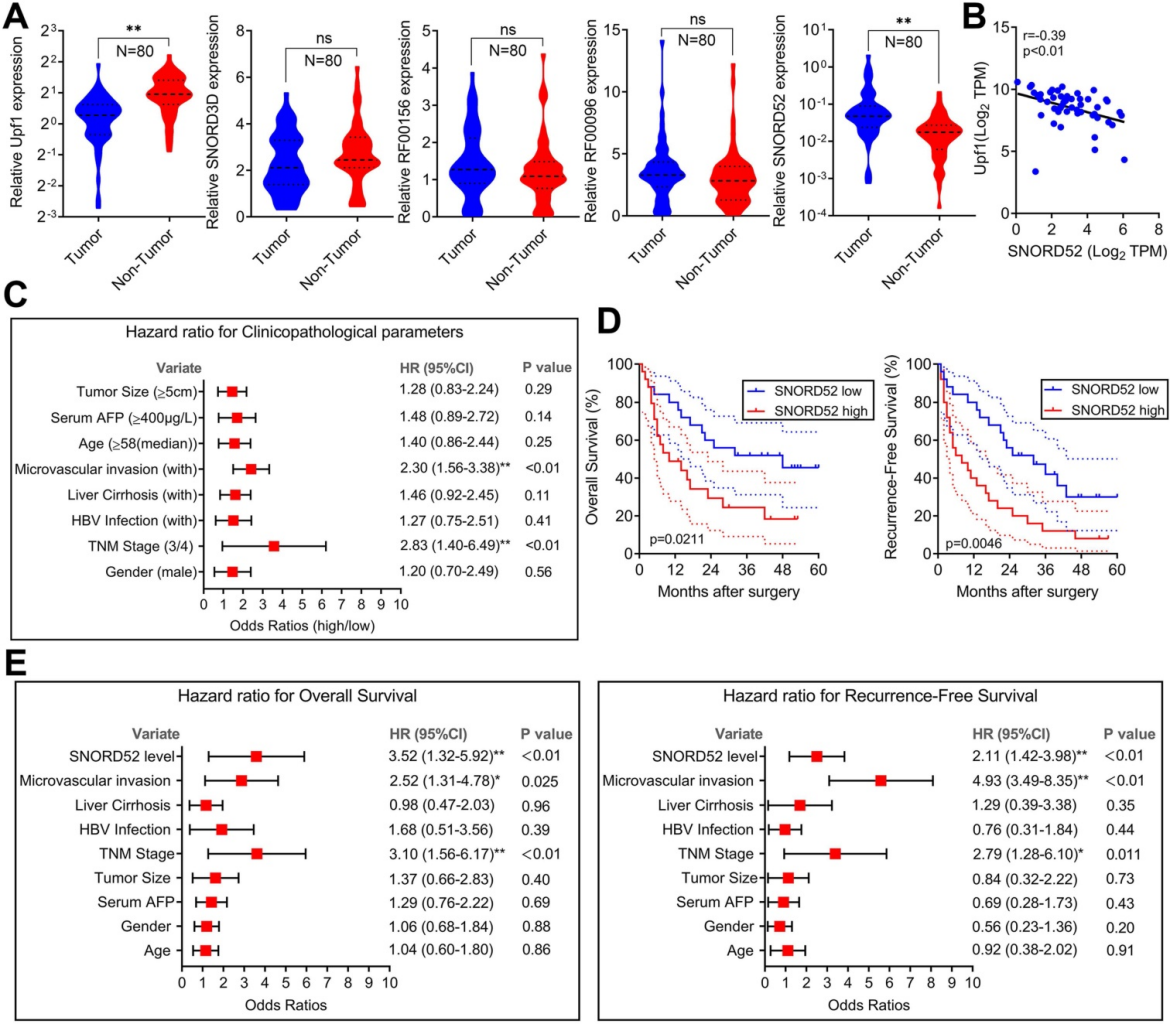
** SNORD52 was upregulated in HCC tissues, negatively correlated with the expression of Upf1 and associated with poor prognosis in HCC. (A)** Quantitative RT-PCR analysis of Upf1, SNORD3D, RF00156, RF00096 and SNORD52 expression in 80 patients with HCC. Upf1 mRNA levels were normalized to GAPDH and snoRNA levels were normalized to U6. Wilcoxon signed-rank test, ns, no significance, *p<0.05, **p<0.01. **(B)** Bivariate correlation analysis of the relationship between Upf1 mRNA and SNORD52 expression levels in HCC tissues. **(C)** Forest plot depicting the correlations between the indicated clinicopathological parameters and the expression levels of SNORD52 (grouped by the median value; χ^2^ test). *p<0.05, **p<0.01. Patients with HCC were divided into the SNORD52 high expression group (whose expression was higher than the median) and the SNORD52 low expression group (whose expression was lower than the median). **(D)** Kaplan-Meier analysis of overall survival and recurrence-free survival based on the SNORD52 expression levels in 80 patients with HCC. The median level of SNORD52 was used as the cutoff. The dashed line indicated 95% confidence interval. **(E)** Forest plot depicting the multivariate analysis by Cox proportional hazards regression model for overall survival and recurrence-free survival.

To explore the role of SNORD52 in determining the clinical status of HCC patients, we detected its expression level in 80 pairs of human HCC tissue samples and pair-matched normal liver tissue samples by quantitative RT-PCR. As shown in **Figure 2A**, Upf1 was significantly downregulated in HCC tissues (*p<0.01*, Wilcoxon signed-rank test), and SNORD52 was significantly upregulated in HCC tissues (*p<0.01*, Wilcoxon signed-rank test). However, there were no significant differences in the expression of SNORD3D, RF00156 and RF00096 between HCC tissues and pair-matched normal liver tissues. The RT-PCR results of SNORD52 expression in HCC are presented in **Figure S3**. Moreover, bivariate correlation analysis showed a significantly negative correlation between SNORD52 and Upf1 expression levels in HCC tissues (*r=-0.39, p<0.01*) (**Figure 2B**). Notably, according to clinical studies, we found that the aberrant expression of SNORD52 was closely correlated with microvascular invasion (*HR=2.30, 95% CI=1.56-3.38, p<0.01*) and TNM stage (*HR=2.83, 95% CI=1.40-6.49, p<0.01*) (**Figure 2C and Table S5**). Furthermore, Kaplan-Meier and log-rank test analyses suggested that HCC patients with high SNORD52 expression had shorter overall survival (*p=0.021*) and poorer recurrence-free survival rates (*p=0.0046*) than those with low SNORD52 expression (**Figure 2D**). Multivariate analysis by the Cox proportional hazards regression model for overall survival and recurrence-free survival showed that the SNORD52 expression level was an independent prognostic risk factor for overall survival (*HR=3.52, 95% CI=1.32-5.92, p<0.01*) and recurrence-free survival (*HR=2.11, 95% CI=1.42-3.98, p<0.01*) (**Figure 2E**). The results demonstrated that SNORD52 could serve as a potential prognostic biomarker in HCC patients.

### SNORD52 knockdown repressed HCC tumorigenesis *in vitro*

**Figure 3:**
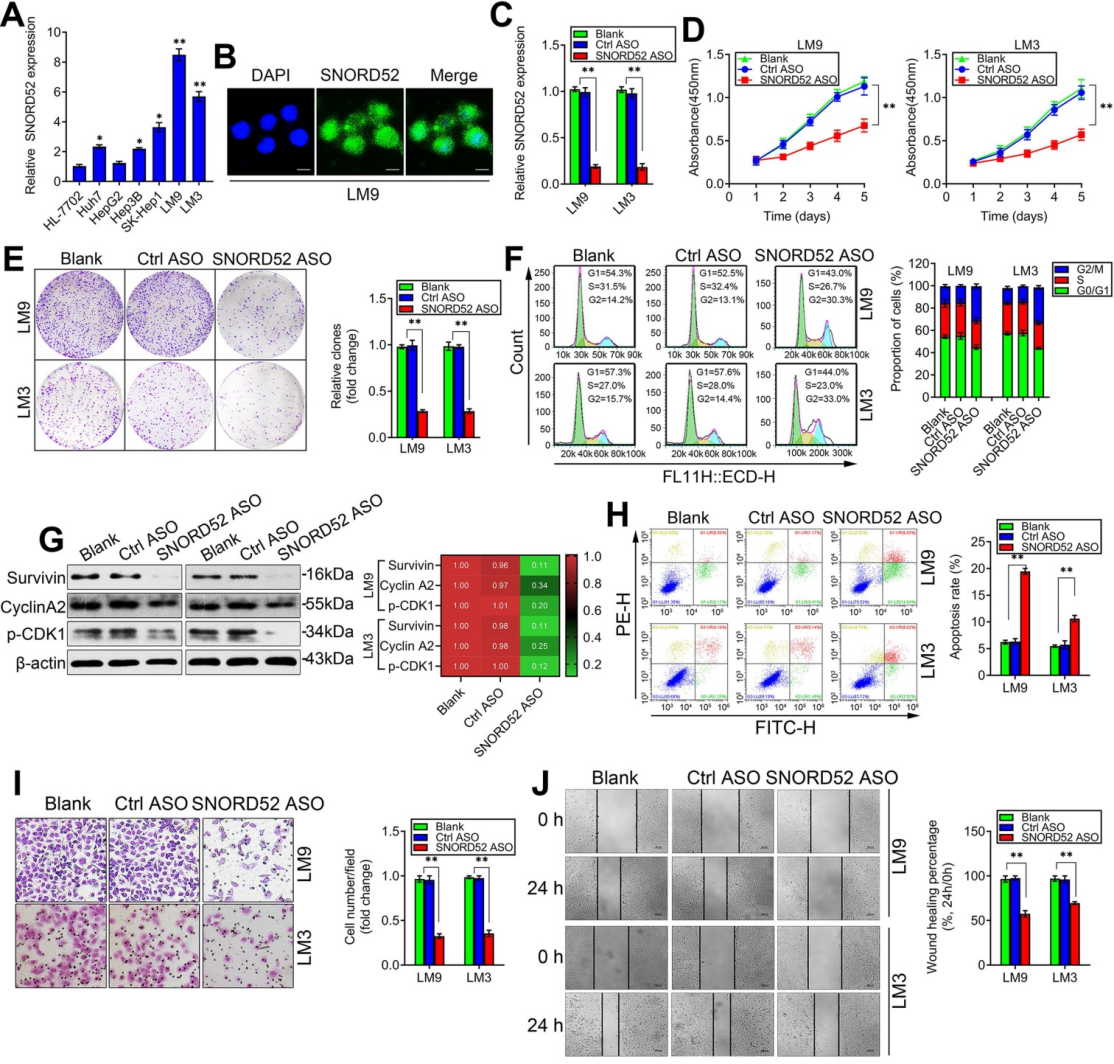
** SNORD52 knockdown repressed HCC tumorigenesis *in vitro*. (A)** Quantitative RT-PCR analysis of SNORD52 expression in the immortalized human hepatic cell line HL-7702 and in HCC cell lines. *p<0.05, **p<0.01 compared with HL-7702 cells.** (B)** Representative FISH images showing the expression of SNORD52 in human HCCLM9 cells. (scale bars=8 µm). **(C)** Quantitative RT-PCR analysis of the SNORD52 expression level after SNORD52 was knocked down in HCCLM9 and HCCLM3 cells by using antisense oligonucleotides (ASOs). *p<0.05, **p<0.01.** (D)** CCK-8 assay revealed that silencing SNORD52 inhibited the proliferation of HCCLM9 and HCCLM3 cells. The data are shown as the mean±S.D. based on at least three independent experiments. *p<0.05, **p<0.01.** (E)** Representative images of colony formation assays of HCCLM9 and HCCLM3 cells after SNORD52 was silenced. *p<0.05, **p<0.01. **(F)** Flow cytometric analysis showing significant decreases or increases in the proportion of cells in the G0/G1, S and G2/M phases; namely, G2/M arrest was induced when SNORD52 was silenced in HCCLM9 and HCCLM3 cells. *p<0.05, **p<0.01. **(G)** Western blotting analysis was performed to evaluate the alterations of the common G2/M phase-related proteins Survivin, p-p53, Cyclin A2 and CDK1.** (H)** Flow cytometric analysis was conducted to evaluate the effects of alterations of the expression of SNORD52 on cell apoptosis in HCCLM9 and HCCLM3 knockdown cells. A significant increase in the percentage of apoptotic cells was observed when SNORD52 was silenced in HCCLM9 and HCCLM3 cells. *p<0.05, **p<0.01.** (I)** Representative images of the cell invasion assay of HCCLM9 and HCCLM3 cells after SNORD52 knockdown. The data are shown as the mean±S.D. based on at least three independent experiments. *p<0.05, **p<0.01. **(J)** Representative images of wound healing assays of HCCLM9 cells and HCCLM3 cells after SNORD52 was silenced for 24 h. All the results were reproducible in three independent experiments. *p<0.05; **p<0.01.

The abnormal upregulation of SNORD52 in HCC tissues suggests that SNORD52 may play an important role in HCC carcinogenesis. To explore the potential biological function of SNORD52, we first analyzed the expression profile of SNORD52 in HCC cells. SNORD52 levels were relatively higher in HCC cell lines (Huh7, HepG2, Hep3B, SK-Hep1, HCCLM9 and HCCLM3) than in HL-7702 cells, which are immortalized, normal human hepatic cells (**Figure 3A**). Next, we chose HCCLM9 and HCCLM3 cells as a knockdown model for subsequent experiments based on their SNORD52 expression levels. Using an RNA FISH assay, we found that SNORD52 was mainly localized in the nucleus of HCCLM9 cells (**Figure 3B**). To obtain a better knockdown effect, antisense oligonucleotides (ASOs) were used to knockdown the SNORD52 level in HCCLM9 and HCCLM3 cells, and the knockdown efficacy was estimated by quantitative RT-PCR (**Figure 3C**). Then, the effects of SNORD52 on the biological behavior of HCC cells were detected using cell biology assays. Functionally, CCK-8 assays showed that SNORD52 knockdown significantly decreased HCC cell proliferation in HCCLM9 and HCCLM3 cells (**Figure 3D**). Colony formation assays showed that SNORD52 knockdown significantly reduced cell proliferation in HCCLM9 and HCCLM3 cell lines (**Figure 3E**). In addition, flow cytometric analysis indicated that SNORD52 knockdown markedly decreased the proportion of cells in the G0/G1 and S phases but increased the proportion of cells in the G2/M phase in HCCLM9 and HCCLM3 cells. In other words, G2/M arrest was induced when SNORD52 was silenced (**Figure 3F**). Consistent with the above data, checkpoint proteins of the G2/M phase, such as Survivin, p-p53, Cyclin A2, and p-CDK1, were downregulated when SNORD52 was silenced in HCCLM9 and HCCLM3 cells (**Figure 3G**). Flow cytometric analysis showed that the proportion of apoptotic cells significantly increased in SNORD52 knockdown HCCLM9 and HCCLM3 cells (**Figure 3H**). The transwell and wound healing assays indicated that SNORD52 knockdown markedly reduced the invasive and migration capability of HCCLM9 and HCCLM3 cells (**Figure 3I-J**). Overall, these data indicated that SNORD52 plays a crucial role in promoting tumorigenesis by facilitating cell motility and cell cycle progression.

### SNORD52 overexpression promoted HCC tumorigenesis *in vitro*

**Figure 4:**
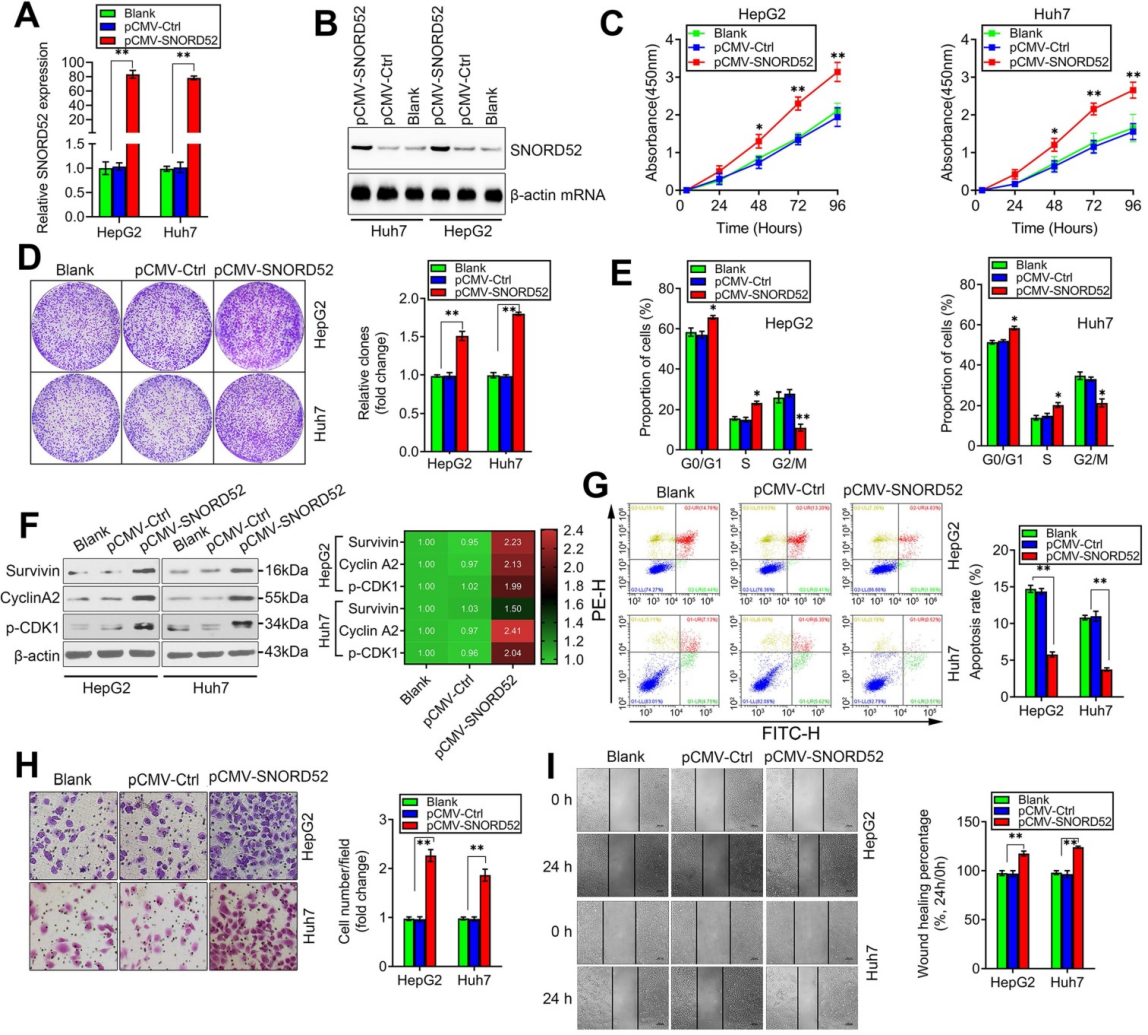
**SNORD52 overexpression promoted HCC tumorigenesis *in vitro*. (A)** SNORD52 overexpression was determined by quantitative RT-PCR in HepG2 and Huh7 cells. The data are shown as the mean±S.D. based on at least three independent experiments. *p<0.05, **p<0.01. **(B)** Northern blot analyses of SNORD52 in HepG2 and Huh7 cells when SNORD52 was overexpressed. **(C)** CCK-8 assay revealed that SNORD52 overexpression promoted the proliferation of HepG2 and Huh7 cells. **(D)** Representative images of colony formation assays of HepG2 and Huh7 cells after SNORD52 was overexpressed. **p<0.01.** (E)** Flow cytometric analysis showing significant increases or decreases in the proportion of cells in the G0/G1, S and G2/M phases; namely, G2/M arrest was inhibited when SNORD52 was overexpressed in HepG2 and Huh7 cells. *p<0.05, **p<0.01.** (F)** Western blotting was performed to evaluate the alterations in the common G2/M phase-related proteins Survivin, p-p53, Cyclin A2 and CDK1 when SNORD52 was overexpressed in HepG2 and Huh7 cells. **(G)** Flow cytometric analysis showed significant decreases in the percentage of apoptotic cells when SNORD52 was overexpressed in HepG2 and Huh7 cells. **p<0.01. **(H)** Representative images of the cell invasion assay of HepG2 and Huh7 cells when SNORD52 was overexpressed. The data are shown as the mean±S.D. based on at least three independent experiments. *p<0.05, **p<0.01.** (I)** Representative images of wound healing assays of HepG2 cells and Huh7 cells after SNORD52 was overexpressed for 24 h. All the results were reproducible in three independent experiments. *p<0.05; **p<0.01.

We next assessed the biological role of SNORD52 by constructing a SNORD52 overexpression model in HepG2 and Huh7 cells that had low endogenous SNORD52 levels using the pCMV-SNORD52 expression plasmid. SNORD52 levels in HepG2 and Huh7 cells drastically increased 48 h after transfection with pCMV-SNORD52 (**Figure 4A**). Northern blot assay further confirmed the above phenomenon (**Figure 4B**). The CCK-8 and colony formation assays implied that SNORD52 overexpression significantly increased HepG2 and Huh7 cell growth (**Figure 4C-D**). Flow cytometric analysis showed that SNORD52 overexpression markedly increased the proportion of cells in the G0/G1 and S phases but decreased the proportion of cells in the G2/M phase in HepG2 and Huh7 cells (**Figure 4E**). The checkpoint proteins of the G2/M phase, such as Survivin, p-p53, Cyclin A2, and p-CDK1, were upregulated when SNORD52 was overexpressed in HepG2 and Huh7 cells (**Figure 4F**). Flow cytometric analysis showed that the overexpression of SNORD52 significantly decreased the proportion of apoptotic HepG2 and Huh7 cells (**Figure 4G**). In addition, the transwell and wound healing assays showed that the overexpression of SNORD52 could significantly increase the ability of invasion and migration in HepG2 and Huh7 cells (**Figure 4H-I**). Overall, the above data further validated the key role of SNORD52 in HCC tumorigenesis.

### SNORD52 promoted HCC tumorigenesis *in vivo*

**Figure 5:**
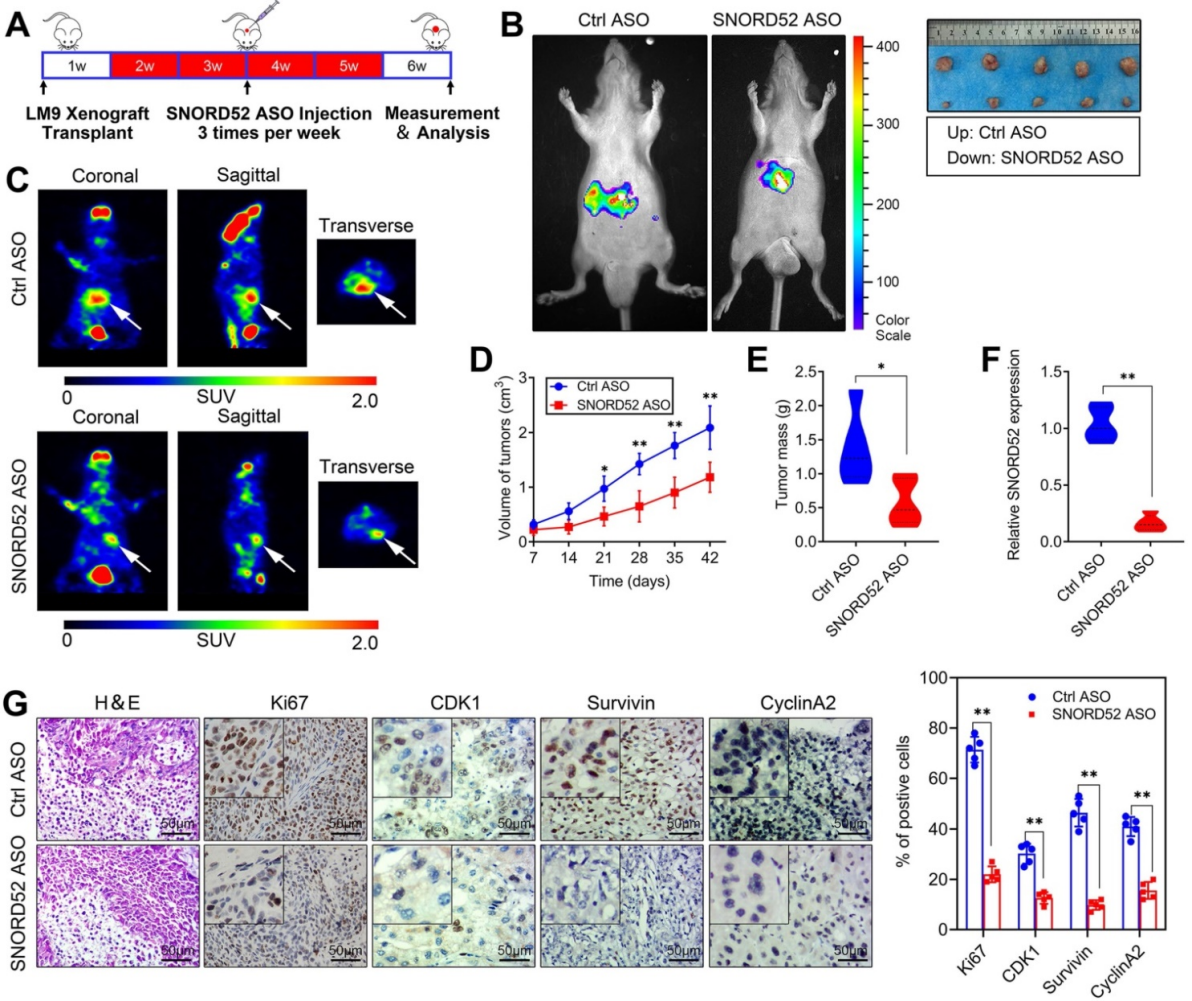
** Effects of SNORD52 on HCC tumorigenesis *in vivo*. (A)** Graphic illustration of the intratumoral injection of *in vivo*-optimized SNORD52 ASOs or control ASOs in the xenograft model. **(B)** Photographs of tumors that developed in xenograft-transplanted nude mouse tumor models viewed with the IVIS Imaging System. Luciferase signals were captured in nude mouse models 5 weeks after injection of SNORD52 ASO or control ASO HCCLM9 cells. Representative images of tumors formed in nude mice injected subcutaneously; the tumors removed from the nude mice are shown. **(C)** Representative images of the PET-CT scans are shown for the SNORD52 depletion and control groups. The photographs were captured in nude mouse models 5 weeks after injection of SNORD52 ASOs or control ASOs. The three different PET-CT layers are shown, and the white arrow shows the tumors. The scale depicts the SUV (standard uptake values) expressed in Bq/ml.** (D)** Effect of SNORD52 knockdown on HCC growth *in vivo* according to the tumor growth curve. The data are shown as the mean±S.D. *p<0.05, **p<0.01. **(E)** Statistical analysis of tumor weight in nude mice. **p<0.01. **(F)** Quantitative RT-PCR analysis of SNORD52 expression levels in SNORD52 ASO-treated HCCLM9 xenograft tumors and the control tumors of HCC xenografts; the data are shown as the mean±S.D. based on at least three independent experiments. **p<0.01.** (G)** Microscopic images of H&E and immunohistochemical staining patterns for Ki67, CDK1, Survivin and Cyclin A2 in the tumor xenografts from nude mice in the SNORD52 knockdown group and the control group. (scale bars=50 µm). *p<0.05, **p<0.01.

Over the years, antisense oligonucleotide (ASO) drugs have received increasing attention due to their ability to target multiple RNAs, and their effects have been verified both *in vitro* and *in vivo* 21-23. To explore the role of SNORD52 in HCC tumorigenesis *in vivo*, we further constructed SNORD52-targeting ASOs and control ASOs with modifications optimized for the *in vivo* study. In brief, HCCLM9 cells were subcutaneously inoculated into the armpits of male nude mice to generate HCC xenografts. After 1 week, the mice were randomly divided into two groups (Control ASO and SNORD52 ASO) and given ASO treatment by intratumoral injection 3 times per week for 4 weeks (**Figure 5A**). Compared to the Ctrl-ASO group, tumor growth was significantly decreased in the SNORD52 group (**Figure 5B**). PET imaging of 18F-fluorodeoxyglucose (18F-FDG) uptake was used to assess the metabolic activity of tumors. The tumors of the SNORD52 ASO group had distinctly decreased metabolic activity compared to those of the control group (**Figure 5C**). Regarding tumor size and mass, SNORD52 ASO significantly reduced overall tumor growth and mass (**Figure 5D-E**). The SNORD52 levels in SNORD52 ASO-treated HCCLM9 tumors of HCC xenografts were significantly downregulated compared to those in the control group (**Figure 5F**). More prominently, the cellular proliferation antigen Ki67 and checkpoint proteins of the G2/M phase were detected in the tumor tissues from xenografts by immunohistochemistry (IHC). All of the above proteins were significantly reduced in SNORD52 ASO-treated HCCLM9 tumors compared with the control group tumors (**Figure 5G**). Taken together, these findings strongly suggest that SNORD52 promotes HCC tumorigenesis *in vivo*.

### SNORD52 combined with CDK1 and increased its protein level by enhancing its stability in HCC cells

**Figure 6:**
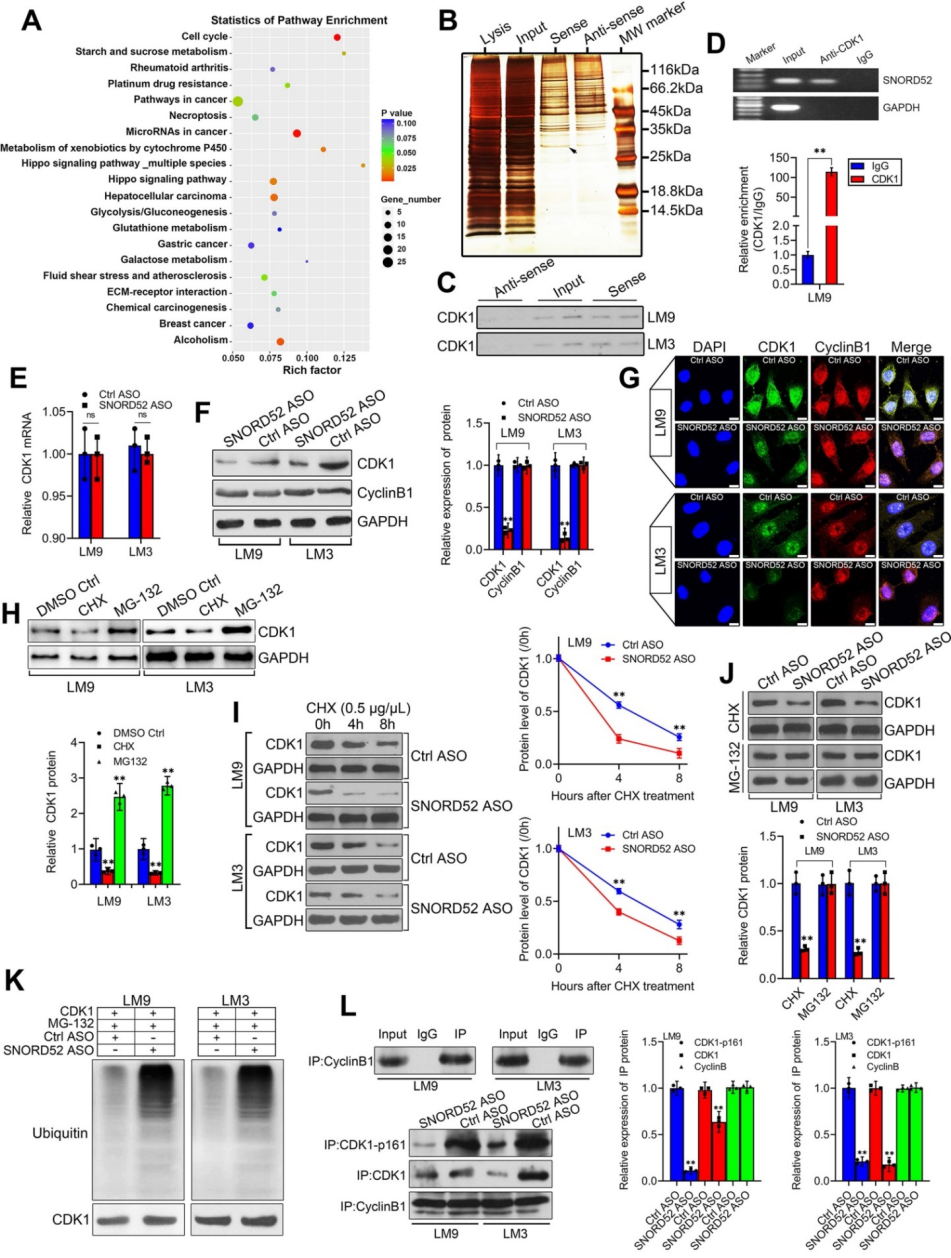
** SNORD52 combined with CDK1 and increased its protein level by enhancing its stability. (A)** KEGG analysis of differentially expressed genes after SNORD52 knockdown; the top 20 pathways are shown.** (B)** Silver-stained SDS-PAGE gel of proteins immunoprecipitated from HCCLM9 cells extracted by SNORD52 and its antisense RNA. The arrow indicates the region of the gel excised for mass spectrum determination by the liquid chromatography dual mass spectrometry method. **(C)** The main proteins in HCCLM9 and HCCLM3 cells found by mass spectrometry analysis were detected by western blotting using the corresponding antibodies. CDK1 proteins were confirmed in the samples pulled down by SNORD52.** (D)** RNA immunoprecipitation (RIP) assays were performed using CDK1 antibody, and specific primers were used to detect SNORD52 with quantitative RT-PCR. **p<0.01.** (E)** CDK1 mRNA levels were detected by using quantitative RT-PCR. The data are shown as the mean±S.D. ns, no significance. **(F-G)** The protein expression levels of CDK1 and Cyclin B1 in HCCLM9 and HCCLM3 cells after SNORD52 knockdown were analyzed by western blotting and immunofluorescence (scale bars=8 µm). **p<0.01. **(H)** Western blotting showed the expression of CDK1 proteins in HCCLM9 and HCCLM3 cells with or without the protein synthesis inhibitor cycloheximide (CHX, 0.5 µg/µL) or the proteasome inhibitor MG-132 (5 µM).** (I)** Western blotting detection of CDK1 levels in HCCLM9 and HCCLM3 cells transfected with Ctrl ASOs or SNORD52 ASOs followed by treatment with CHX (0.5 µg/µL) for the indicated time points. The quantitative analysis is also shown (right panel). The data are shown as the mean±S.D. based on at least three independent experiments. **p<0.01.** (J)** SNORD52 knockdown HCCLM9, HCCLM3 cells and control cells were incubated with CHX (0.5 µg/µL) and MG132 (5 µM) for 24 hours. The levels of CDK1 proteins were detected by western blotting (up panel) and quantified by densitometry (down panel). The experiments were performed in triplicate; the data are expressed as the mean±S.D. **p<0.01.** (K)** SNORD52 prevented CDK1 ubiquitination. Depletion of SNORD52 enhanced CDK1 ubiquitination. HCCLM9 and HCCLM3 cells were transfected with SNORD52 ASO and 48 h post-transfection, cells were treated with MG132 for 6 h and then cells were subjected to Western blot analysis using an anti-CDK1 or an anti-ubiquitin antibody.** (L)** Co-IP assays indicated that SNORD52 knockdown significantly decreased the interaction between p-CDK1 and Cyclin B1.

To better understand the underlying mechanism of SNORD52 in HCC tumorigenesis, first, KEGG analysis of differentially expressed genes was used to find the signaling pathways SNORD52 regulated, and the result implied that SNORD52 could affect the expression of genes involved in the cell cycle (**Figure 6A**). Recent studies have shown that many RNAs participate in molecular regulatory pathways through interactions with proteins. To explore whether SNORD52 works in this way, RNA pulldown assays were performed to identify proteins associated with SNORD52 in HCC cells. Specific bands of SNORD52 were excised and analyzed by mass spectrometry (**Figure 6B and Table S6**). Cyclin-dependent kinase 1 (CDK1) was identified by mass spectrometry as the major protein combined with SNORD52 (**Figure S4**). CDK1 was detected by western blotting in RNA pull-down experiments (**Figure 6C**). Moreover, RNA immunoprecipitation (RIP) was also performed using HCCLM9 cell extracts with antibodies against CDK1. The enrichment of SNORD52 was observed, but no GAPDH mRNA enrichment was observed (**Figure 6D**). The above results were consistent with the results of the cell function and KEGG enrichment analyses. As CDK1 mainly binds to Cyclin B1 to play a key role in regulating the cell cycle 24, to illustrate the effect of SNORD52 on CDK1, the expression levels of CDK1 mRNA and protein and Cyclin B1 protein in SNORD52 knockdown HCCLM9 and HCCLM3 cells were detected. The results indicated that SNORD52 affected CDK1 protein levels but not mRNA levels or Cyclin B1 protein levels in HCC cells (**Figure 6E-G**). Based on the above findings, we speculate that SNORD52 binding to CDK1 and affecting its biological activity may occur posttranscriptionally. To confirm these hypotheses, CDK1 protein levels were detected in HCCLM9 and HCCLM3 cells treated with the protein synthesis inhibitor cycloheximide (CHX) or the proteasome inhibitor MG-132, with dimethyl sulfoxide (DMSO) as the control reagent. The results showed that CHX downregulated CDK1 protein, while MG-132 upregulated CDK1 protein (**Figure 6H**). In addition, using CHX assays, we found that the half-life of CDK1 protein was shortened in SNORD52 knockdown HCCLM9 and HCCLM3 cells (**Figure 6I**). Then, SNORD52 knockdown HCCLM9 and HCCLM3 cells were treated with CHX or MG-132. The results showed that MG-132 could eliminate the changes in CDK1 protein levels in SNORD52 knockdown HCCLM9 and HCCLM3 cells, while CHX did not have this effect (**Figure 6J**). Moreover, knockdown of endogenous SNORD52 in HCCLM9 and HCCLM3 cells increased levels of endogenous CDK1 ubiquitination (**Figure 6K**). These data suggested that SNORD52 stabilized CDK1 protein by blocking its ubiquitination and proteasomal degradation *in vitro*. In addition, Co-IP assays indicated that SNORD52 knockdown significantly decreased the interaction between p-CDK1 and Cyclin B1 (**Figure 6L**). In summary, our data indicate that SNORD52 reduces the degradation rate of the CDK1 protein and enhances its stability and phosphorylation level in HCCLM9 and HCCLM3 cells.

### The biological function of SNORD52 was dependent on the presence of CDK1

CDK1 is a member of the cell cycle-dependent kinase (CDK) family and belongs to the serine/threonine kinase family, playing a vital role in regulating the cell cycle 25-28. Using the TCGA database (https://www.cancer.gov/) and GEPIA2 database (http://gepia2.cancer-pku.cn/) 29, we found that CDK1 was generally upregulated in digestive tumors (liver hepatocellular carcinoma, cholangiocarcinoma, pancreatic adenocarcinoma, stomach adenocarcinoma, esophageal carcinoma and colon adenocarcinoma) (**Figure S5A**) and closely related to the poor prognosis of hepatobiliary and pancreatic tumors (**Figure S5B-C**). To further investigate the effect of CDK1 on the function of SNORD52, rescue assays were conducted. We overexpressed CDK1 in SNORD52 knockdown HCCLM9 and HCCLM3 cells, and proliferation, invasion, migration and the cell cycle were measured. The results showed that the effects of SNORD52 ASO on the proliferation, colony formation, invasion, migration and cell cycle of HCCLM9 and HCCLM3 cells can be offset by CDK1 overexpression (Fi**gure 7A-G**). These results were reversed when knockdown CDK1 in SNORD52 overexpressed HepG2 and Huh7 cells. (**Figure S6**). Furthermore, CDK1 was significantly upregulated in HCC tissues and bivariate correlation analysis showed a significantly positive correlation between SNORD52 and CDK1 or checkpoint proteins of the G2/M phase (Survivin and CyclinA2) expression levels in HCC tissues. (**Figure S7**). In summary, the above data indicate that the biological role of SNORD52 is partly dependent on the presence of CDK1 and higher SNORD52 promoted CDK1 signaling.

## Discussion

For many years, hepatocarcinogenesis has been considered to be a multistage process, including genetic and epigenetic changes as well as external microenvironmental factors, which eventually lead to the malignant transformation of liver cells 30. At present, due to the lack of ideal diagnostic biomarkers for HCC, the treatments for HCC patients are very limited, so once HCC patients are diagnosed, most of them are in intermediate and advanced stages. More seriously, postoperative recurrence is mainly caused by intrahepatic and extrahepatic metastases, which are also the main reasons for the poor prognosis of HCC patients. Until now, there is still much to be done to achieve effective interventions for hepatocarcinogenesis. Our previous research showed that Upf1, as a tumor suppressor gene, plays an important role in hepatocarcinogenesis 19. To identify the noncoding RNA regulated by Upf1 responsible for hepatocarcinogenesis, in the present study, we conducted high-throughput RNA-seq based on an Upf1 knockdown cell model. Combining sequencing data with abnormally expressed genes in HCC tissues, we identified SNORD52 as an Upf1-regulated snoRNA. SNORD52 potentially exhibited an oncogenic effect in HCC. In addition, according to clinical data, the level of SNORD52 was proven to be a prognostic factor for HCC for the first time.

snoRNAs have been regarded as the class of ncRNAs with the best characteristics. In vertebrates, except for a few snoRNAs transcribed autonomously by RNA polymerase II, most snoRNAs are encoded in introns of protein-coding or noncoding genes 10, 31. The biogenesis of most intron snoRNAs includes cooperative transcription with host genes, the splicing of intron lariats and nucleolytic digestion in the nucleoplasm 32, 33. Previous studies have suggested that snoRNAs function as housekeeping genes in cells. Recent studies have shown that snoRNAs have tumor suppressor or carcinogenic functions in many types of cancers and are involved in a number of biological cancer processes, including cell death, the activation of invasion and metastasis, angiogenesis, and continuous proliferation signaling 34. Numerous studies have shown that many snoRNAs are stable and detectable in body fluids, including cancer patients' plasma, serum, and urine. Their expression levels are closely related to diagnosis, prognosis and the classification of subtypes 35-37. Given these properties, snoRNAs have the potential to become cancer biomarkers. In this study, we provided the first evidence of SNORD52 dysregulation in HCC. Specifically, the upregulation of SNORD52 was observed in 80 HCC tissues and in different hepatoma cell lines. Moreover, SNORD52 expression was closely associated with microvascular invasion and TNM stage and potentially correlated with HCC formation and progression. Multivariate analyses by Cox proportional hazards regression models for overall survival and recurrence-free survival showed that the SNORD52 expression level was an independent prognostic risk factor for overall survival and recurrence-free survival. Due to the low abundance of SNORD52 expression in tissues, we tried to detect the expression of SNORD52 in the blood of HCC patients in the preliminary experiments of this study, but it was not found. We plan to detect the expression of SNORD52 in the blood using RNA sequencing in future research. Moreover, in our study, SNORD52 had higher expression levels in LM9 and LM3 cells, which was a similar expression pattern as Upf1. Both Upf1 and SNORD52 were at high expression levels in LM9 and LM3. However, as show in **Figure 1E-F**, in the same cell line, Upf1 knockdown lead to up-regulation of SNORD52. The above data was consistent with RNA-sequencing. The negative correlation between Upf1 and SNORD52 was further conformed in clinical HCC samples which had more clinically value. We speculate that Upf1 protein may be regulated by certain post-transcriptional factors in some HCC cell lines and we will continue to explore its possible mechanisms in the future study.

Protein-coding RNAs and noncoding RNAs (ncRNAs) are expressed in human cells through a complex coordinated network. Some genes simultaneously encode mRNAs and regulatory small RNAs from the same master transcript, which raises the question of how the difference between encoding RNAs and ncRNAs is achieved from this single precursor 10, 38. Research has shown that the nonsense-mediated decay (NMD) quality control pathway is the key to solving this problem by selectively degrading specific alternatively processed RNAs. Some genes contain multiple different snoRNAs, sometimes even within the same intron. These genes are widely alternatively spliced, often producing spliced RNAs that are degraded by NMD. By adjusting the splicing mode, the cell could ensure that only specific snoRNAs are generated. In addition to NMD, the RNA decay mechanism may also affect the output of other multicomponent genes 39. Studies have shown that snoRNA host genes (SNHGs) are regulated by NMD. NMD is at the forefront of regulating snoRNA production 40. As the most important trans-acting factor of NMD, Upf1 plays an important role in NMD 41. Our previous studies showed that promoter methylation caused Upf1 downregulation in HCC and that Upf1 regulated hepatocarcinogenesis and acted as a tumor suppressor gene in HCC. Upf1 regulated the expression of snoRNA host gene 6 (SNHG6) in HCC 19, 42. To the best of our knowledge, these were the first reports about Upf1-mediated NMD dysregulation in HCC. Based on the above findings, in this study, we explored the relationship between Upf1 and snoRNAs. Using high-throughput RNA sequencing and clinical specimens, we identified SNORD52 as one of the snoRNAs regulated by Upf1. The result was further verified at the cellular level. The above results were consistent with the findings of previous studies; that is, Upf1-mediated NMD regulates snoRNA expression levels to a certain extent in HCC. During the hepatocarcinogenesis, methylation of the promoter region leads to the downregulation of Upf1 which was an important transcription factor in the NMD pathway. The progress of NMD was suppressed and the premature termination codon (PTC) and exon junction complex (EJC) generated during the transcription of SNORD52 could not be recognized and degraded in time and eventually caused the upregulation of SNORD52. As mentioned above, the detailed mechanisms by which NMD regulates snoRNA expression are complex, and many mechanisms are not yet clear. Elucidating this activity was not the aim of the current study but warrants further research. We will explore the possible mechanism by which Upf1 regulates SNORD52 in future work.

**Figure 8:**
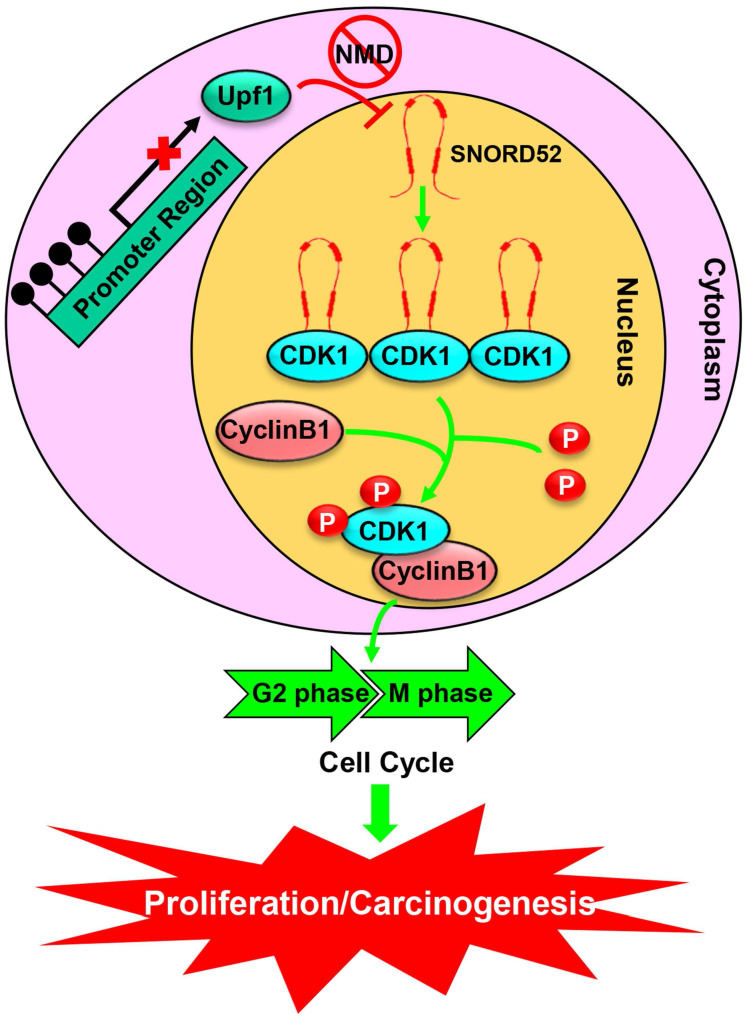
** A schematic model of SNORD52 functions during hepatocarcinogenesis.** Methylation of the promoter region leads to the downregulation of Upf1, inhibiting the progression of NMD and causing the upregulation of SNORD52. SNORD52 promotes the cell cycle by binding to CDK1, increasing its protein or phosphorylation levels. Ultimately, this leads to HCC carcinogenesis.

To further understand the biological function of SNORD52 in HCC progression, we investigated the malignant features of SNORD52 in HCC cell lines using gain-of-function and loss-of-function experiments. We demonstrated that SNORD52 exhibited an oncogenic effect in HCC both *in vitro* and *in vivo*. Antisense oligonucleotides are stable in the blood, and recent studies have demonstrated their ability to silence *in vivo* noncoding RNAs, indicating potential applications for cancer therapy 21, 43, 44. Consistent with previous data, in this study, using the method of intratumoral injection of ASOs, we silenced the expression of SNORD52 *in vivo*. We believe that targeting a single snoRNA molecule (such as SNORD52) and clearly establishing its molecular function in human HCC cells may be a more powerful and specific means of achieving therapeutic goals. Using an RNA pull-down assay and RIP assay, cyclin-dependent kinase 1 (CDK1), the key G2/M check-point protein in the cell cycle, was identified as the binding protein of SNORD52. We also found that SNORD52 could increase CDK1 levels by enhancing the stability of CDK1 proteins and that the function of SNORD52 was dependent on the presence of CDK1 protein. In addition, according to the analysis of the RNA pulldown and mass spectrometry, SNORD52 may also bind other proteins (Table S6). Some proteins such as VIM (Vimentin), FUS and NOP2 were closely related to cell invasion. The effect of SNORD52 on promoting invasion may depend on the above proteins and warrants further study. Combined with our previous studies, these highly consistent data clarified that the Upf1/SNORD52/CDK1/cell cycle gene pathway is involved in promoting HCC tumorigenesis (**Figure 8**).

## Conclusion

In summary, the present study characterized the snoRNA expression profiles in HCC and identified SNORD52 as an Upf1-regulated snoRNA. SNORD52 was upregulated in HCC tissues and was closely associated with the poor prognosis of patients with HCC, acting as a functionally relevant snoRNA in HCC. In addition, a series of *in vitro* and *in vivo* experimental results supported the mechanistic role of SNORD52 in HCC tumorigenesis. Mechanistically, SNORD52 promoted hepatocarcinogenesis by binding to CDK1, increasing its protein or phosphorylation levels. The newly discovered Upf1/SNORD52/CDK1 signaling pathway is involved in hepatocarcinogenesis and targeting this pathway may provide new therapeutic targets for HCC treatment.

## Supplementary Material

Supplementary figures and tables.

## Figures and Tables

**Figure 7:**
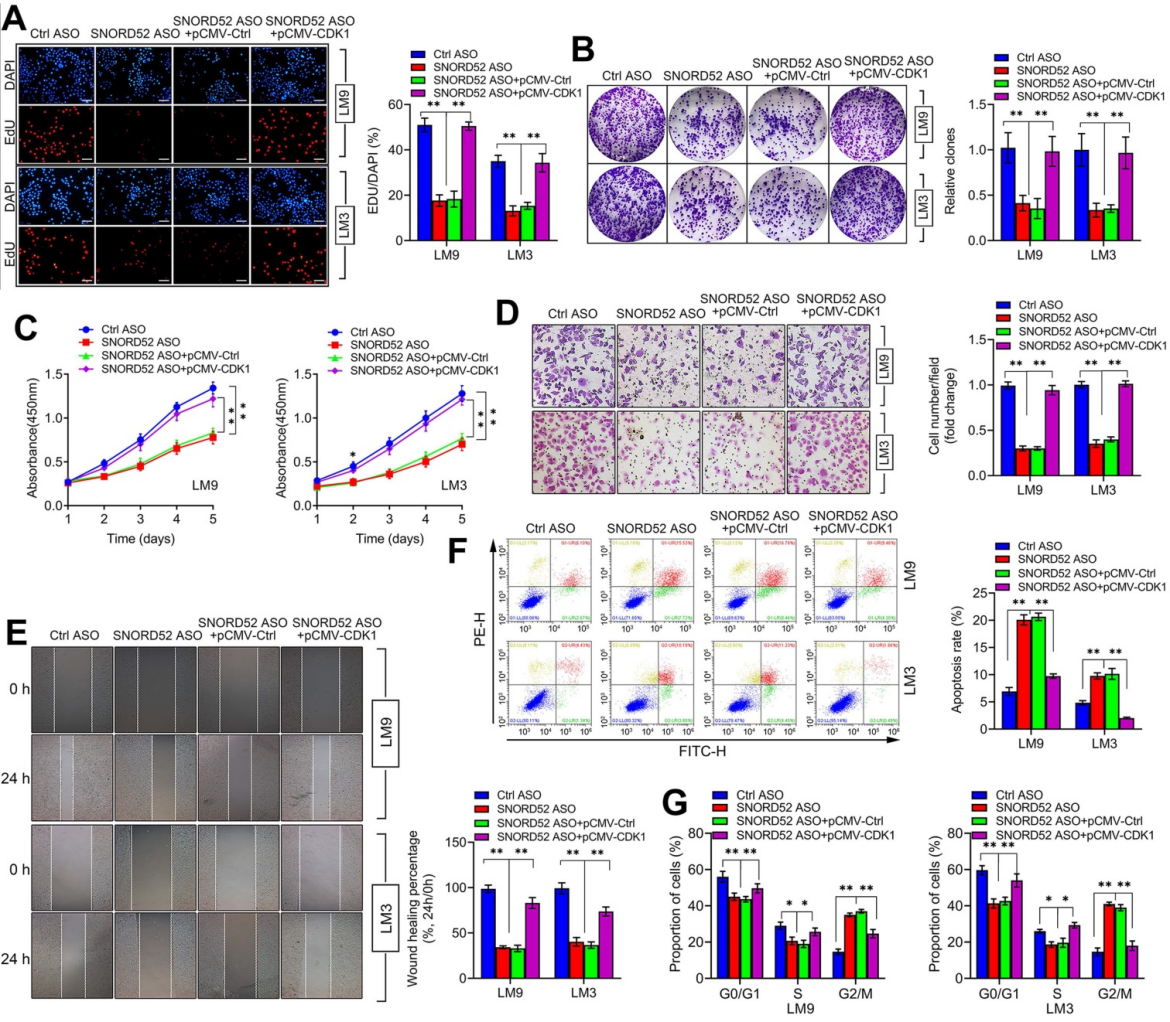
** The biological function of SNORD52 was dependent on the presence of CDK1.** pCMV-CDK1 and control pCMV were transfected into SNORD52 knockdown HCCLM9 and HCCLM3 cells. **(A)** The cell proliferation rate was assessed using EdU assays. *p<0.05, **p<0.01.** (B)** Colony formation assays were conducted to evaluate the proliferation ability of SNORD52-silenced HCCLM9 and HCCLM3 cells when pCMV-CDK1 and control pCMV were transfected. *p<0.05, **p<0.01.** (C)** CCK-8 assays showed that the upregulation of CDK1 weakened the effect of SNORD52 on HCCLM9 and HCCLM3 cell proliferation. *p<0.05, **p<0.01. **(D)** Transwell assays showed that the upregulation of CDK1 weakened the effect of SNORD52 on HCCLM9 and HCCLM3 cell invasion.** (E)** Wound healing assays showed that the upregulation of CDK1 weakened the effect of SNORD52 on HCCLM9 and HCCLM3 cell migration.** (F)** Upregulation of CDK1 weakened the effect of SNORD52 on HCCLM9 and HCCLM3 cell apoptosis**. (G)** Upregulation of CDK1 weakened the effect of SNORD52 on HCCLM9 and HCCLM3 cell cycle progression. *p<0.05, **p<0.01.

## Abbreviations

HCC: hepatocellular carcinoma
Upf1: up-frameshift 1
LncRNA: long noncoding RNA
snoRNA: small nucleolar RNA
OS: overall survival
RFS: recurrence-free survival
NMD: nonsense-mediated mRNA decay
RT-PCR: reverse transcription PCR
RT-qPCR: reverse transcription quantitative PCR
FISH: fluorescence *in situ* hybridization
ASO: antisense oligonucleotide
mRNA: messenger RNA
cDNA: complementary DNA
siRNA: small interfering RNA
SDS-PAGE: sodium dodecyl sulfate-polyacrylamide gel electrophoresis
CCK-8: Cell Counting Kit-8
Edu: 5-ethynyl-2'-deoxyuridine
CDK1: Cyclin-dependent kinase 1
18F-FDG: 18F-fluorodeoxyglucose
IHC: immunohistochemistry
RIP: RNA immunoprecipitation
DMSO: dimethyl sulfoxide
CHX: cycloheximide
