# Supplementary material for: The C/D box small nucleolar RNA SNORD52 regulated by Upf1 facilitates Hepatocarcinogenesis by stabilizing CDK1
Source: Theranostics. 2020 Jul 23;10(20):9348–63. doi: 10.7150/thno.47677 (PMC7415794; doi:10.7150/thno.47677)
Supplement: Supplementary file 1 — Supplementary figures and tables. [file thnov10p9348s1.pdf]

Supplementary Information

The C/D box small nucleolar RNA SNORD52 regulated by Upf1 facilitates hepatocarcinogenesis by stabilizing CDK1

Running title: SNORD52 exhibits an oncogenic effect in HCC

Cuicui Li[1], Long Wu[2], Pengpeng Liu[2], Kun Li[2], Zhonglin Zhang[2], Yueming He[2], Quanyan Liu[2], Ping Jiang[2], Zhiyong Yang[2], Zhisu Liu[2], Yufeng Yuan[2]⌧, Lei Chang[2]⌧

1. Department of Integrated Internal Medicine and Geriatrics, Zhongnan Hospital of Wuhan University, Wuhan 430071, P.R. China.
2. Department of Hepatobiliary and Pancreatic Surgery, Zhongnan Hospital of Wuhan University, Wuhan 430071, P.R. China.

⌧ **Corresponding Authors:** Lei Chang, M.D., Ph.D., Department of Hepatobiliary and Pancreatic Surgery, Zhongnan Hospital of Wuhan University, #169 Donghu Road, Wuhan 430071, P.R. China. Tel.:+86 13237102029; E-mail: reniorchang@whu.edu.cn. Yufeng Yuan, M.D., Ph.D., Department of Hepatobiliary and Pancreatic Surgery, Zhongnan Hospital of Wuhan University, #169 Donghu Road, Wuhan 430071, P.R. China. Tel.:+86 13995564795; E-mail: yuanyf1971@163.com.

Supplementary Experimental Procedures

RNA-sequencing (RNA-seq) analysis

Total RNA was extracted using Trizol reagent (Invitrogen, CA, USA) following the manufacturer's procedure. The total RNA quantity and purity were analysis of Bioanalyzer 2100 and RNA 6000 Nano LabChip Kit (Agilent, CA, USA) with RIN number >7.0 [1]. RNA degradation and contamination was monitored on 1% agarose gels. RNA purity was checked using the NanoPhotometer® spectrophotometer (IMPLEN, CA, USA). RNA concentration was measured using Qubit®RNA Assay Kit in Qubit® 2.0 Flurometer (Life Technologies, CA, USA). RNA integrity was assessed using the RNA Nano 6000 Assay Kit of the Bioanalyzer 2100 system (Agilent Technologies, CA, USA). Sequencing libraries were generated using NEBNext® Ultra™ RNA Library Prep Kit for Illumina® (NEB, USA) following manufacturer's recommendations. First strand cDNA was synthesized using random hexamer primer and M-MuLV Reverse Transcriptase (RNase H-). Second strand cDNA synthesis was subsequently performed using DNA Polymerase I and RNase H. The library quality was assessed on the Agilent Bioanalyzer 2100 system. The clustering of the index-coded samples was performed on a cBot Cluster Generation System using TruSeq PE Cluster Kit v3-cBot-HS (Illumia) according to the manufacturer's instructions. After cluster generation, the library preparations were sequenced on an Illumina Hiseq 2500 platform and 125 bp paired-end reads were generated. Reference genome and gene model annotation files were downloaded from genome website browser (NCBI/UCSC/Ensembl) directly. Indexes of the reference genome were built using Bowtie v2.0.6 [2], and paired-end clean reads were aligned to the reference genome using TopHat v2.0.9 [3]. HTSeq v0.6.1 was used to count the read numbers mapped of each gene. Differential expression analysis between two groups was performed using the DESeq R package

based on the negative binomial distribution. The resulting P values were adjusted using the Benjamini and Hochberg's approach for controlling the False Discovery Rate (FDR). Genes with |log2[fold change (FC)]| > 1 and FDR < 0.05 found by DESeq were assigned as differentially expressed [4]. RIP-seq raw reads were mapped to the human reference genome (hg38/GRCh38).

Fluorescence in situ hybridization (FISH)

RNA FISH was used to observe the relative subcellular localization and molecular abundance. FISH was performed according to the Ribo Fluorescence In Situ Hybridization Immobilized Kit (RN: 10910; RiboBio Co., Ltd. Guangzhou, China) protocol. After prehybridization buffer treatment, the probe mixture was diluted in hybridization buffer after removing prehybridization buffer and was incubated overnight at 37 ℃. The DNA was dyed with DAPI for 10 minutes before sealing. The subcellular localization and molecular abundance were observed under the same optical conditions with a Double Disc Laser Confocal Imaging System (UltraVIEW VOX & 1 × 81; Perkin Elmer & Olympus).

Cell proliferation assay

Cell proliferation assays were conducted using a Cell Counting Kit-8 (Dojindo Molecular Technologies Inc., Kumamoto, Japan) according to the manufacturer's protocol. Hepatoma cells were plated in 24-well plates in triplicate at a density of 2-5 $\times 10^4$ cells per well and cultured in growth medium. Cells were treated with the ASOs or plasmids, and the numbers of cells per well were measured by the (450 nm) at the indicated time points. Additionally, for the colony formation assay, 500 cells were seeded in 6-well plates for 10 days, and colonies were fixed and stained with crystal violet solution. Each cell line was evaluated in three parallel replicates.

Flow cytometric analysis

For cell cycle analyses, HCCLM9 or HCCLM3 cells were harvested 48 h after transfection with siRNA, ASOs and vector, washed with phosphate-buffered saline (PBS), and fixed in 75% ethanol at 4 ℃ overnight. RNA was removed from the preparations by incubating the cells with RNase A (Sigma-Aldrich) at 37 ℃ for 30 min. Cells were then stained with propidium iodide (PI) solution (Sigma-Aldrich) for 30 min at room temperature and analyzed on a FACSAria I flow cytometer (BD Biosciences). Apoptosis was assessed 48 h after transfection using a FITC Annexin V Apoptosis Detection Kit I (BD Pharmingen, San Diego, CA, USA) and an Accuri C6 Flow Cytometer (BD Biosciences).

Western blot analysis

Cellular proteins from each sample were separated by SDS-polyacrylamide gel electrophoresis (4% stacking and 10% separating gels) and then transferred onto polyvinylidene fluoride (PVDF) membranes (Millipore, USA). The membranes were then incubated with primary antibodies overnight at 4 ℃. After the membranes were incubated with secondary antibodies, they were subsequently subjected to immunoblot analysis using an ECL immunoblotting kit (Beyotime Institute of Biotechnology, China) according to the manufacturer's protocol. The antibodies used in this study are listed in Table S2.

Transwell assay

The invasion of cells was assessed using Matrigel-coated chambers with 8-μm pores (BD Biosciences, Franklin Lakes, NY, USA). Briefly, hepatoma cells (1×10^5) were seeded in serum-free medium and were allowed to translocate toward complete media supplemented with 10% fetal bovine serum after knockdown of lncRNA-SNHG6. The cells that had invaded through the membrane to the lower surface were fixed, stained and counted after 24 h.

Colony formation assay

For soft agar colony formation assay, forty-eight hours after transfection with the indicated siRNAs, vector or ASOs, HCC cells were suspended in DMEM containing 10% FBS and 0.3% Seaplaque low melting temperature agarose (Lonza), and 1.5 ml agarose containing 5×10^3 cells were plated in one well of 6-well plates over a 1.5 ml layer of DMEM/10% FBS/0.6% agarose. Cells were incubated at 37 ℃ for 2 weeks, before they were fixed and stained with trypan blue. The colonies were then scored under microscope.

Hematoxylin-eosin (HE), Immunohistochemistry (IHC) and Immunofluorescence (IF)

For the HCC and adjacent tissue samples, tissue sections were deparaffinized in xylene and rehydrated with ethanol before paraffin embedding. All the tissue samples were sectioned to produce 4-mm thick slices. To perform HE staining, slices were stained with hematoxylin and eosin for 3 minutes and 5 seconds after dewaxing. For IHC, paraffin sections were cut to a thickness of 4 μm, the slides were deparaffinized in xylene and rehydrated with ethanol, and the endogenous peroxidase was inactivated with 0.3% hydrogen peroxide. All of the steps were performed using an UltraSensitiveTM S-P kit (Maixin, Fuzhou, China) according to the manufacturer's protocol. The total immunostaining score was calculated as the sum of the positive percentage and the staining intensity of the stained cells, which ranged from 0 to 6. The percent positivity was scored as "0" (0-25%), "1" (26-50%), "2" (51-75%), and "3" (≥ 75%). The staining intensity was scored as "0" (no staining), "1" (weakly stained), "2" (moderately stained), and "3" (strongly stained). Negative expression of protein was defined as a total score ≤3, and positive expression was defined as a total score ≥4. For immunofluorescence, cells were fixed in 4% paraformaldehyde, permeabilized using 0.5% Triton X-100 and incubated with primary antibody and secondary antibodies according to the

manufacturer's protocol. The coverslips were counterstained with DAPI and imaged with a confocal laser scanning microscope (Olympus FV1000). The antibodies used in this study are listed in Table S2.

5-ethynyl-2'-deoxyuridine (EdU) assay

EdU (5-ethynyl-2'-deoxyuridine), a nucleoside analog of thymidine, is readily incorporated into cellular DNA during DNA replication. Cell proliferation was evaluated using a Cell-Light EdU Apollo 567 In Vitro Imaging Kit (RiboBio, Guangzhou, China) as described by the manufacturer. Briefly, cells were incubated with 50 μM EdU for 2 h at 37 °C, fixed with 4% formaldehyde, stained with Apollo reaction cocktail and Hoechst 33342, and protected from light. Images were acquired by fluorescence microscopy and overlapped using Image-Pro Plus software (Version 6.0.0.260; Media Cybernetics, Inc., Tokyo, Japan).

Wound healing assay

HCC cells (1×10^6 cells/well) were treated with the indicated reagents, and wounds were made using a 100-μl plastic pipette tip. The size of the wound was measured after 24 h of wound formation and imaged. The cell migration area was measured between dashed regions using ImageJ software (NIH, Bethesda, MD) and normalized to control cells.

Northern blot analyses

Total RNA extracted from extracted from HCC cells with the Trizol reagent (Invitrogen). Briefly, 10 μg RNA was denatured in loading buffer, resolved on a 1% agarose gel, and transferred to a Hybond N nylon membrane (Amersham Bioscience Co., Piscataway, NJ, USA) by electrophoresis using a semidry transfer cell (Bio-Rad). Then, the membrane was prehybridized in DIG Easy Hyb (Roche, Indianapolis, IN, USA). Subsequently, a specific probe against SNORD52 (nucleotide position 1-50)

was labelled using the DIG-High Prime DNA Labeling and Detection Starter Kit II (Rocahe, USA) according to manufacturer's instructions. Finally, the membrane was washed and signals detected using a Bioimaging Analyzer GelDoc XR (Bio-Rad). The size of each transcript was determined by comparing its corresponding band to the low range ssRNA ladder (New England Biolabs, #N0364S). The β-actin mRNA was used as an internal control. The primers used for this analysis were as follows: primer-SNORD52: forward 5'-GGGAATGATGATTTCACAGACT-3', reverse 5'-TGACA TCATGACCAGCATCG-3', and primer-β-actin: forward 5'-AGAGCTACGAGCTGCCTGAC-3', reverse 5'-AGCACTGTGTTGGCGTACAG-3'.

Animal Model

The animal studies were approved by the Institutional Animal Care and Use Committee (IACUC) of Wuhan University in Wuhan, China. Male athymic 4-week-old BALB/c nude mice were obtained from the Animal Center of the Chinese Academy of Medical Sciences (Beijing, China) and were maintained in a specific pathogen-free facility. For xenograft implantation experiments, HCCLM9 cells were harvested from 6-well plates and suspended at 3-5 $\times 10^6$ cells/ml. The suspended cells (100 μl) were subcutaneously injected into the armpits of 10 mice (5 mice per group). After 1 weeks, mice were randomly divided into two groups (Control ASO and SNORD52 ASO) and given respective ASO treatment (5 nmol per injection, RiboBio, Guangzhou, China) by intratumor injection 3 times per week for 4 weeks. When the study finished, the mice were anesthetized in 6 weeks, and the tumor volume and weight were measured. Bioluminescence imaging and tumor dissection were performed as described [5].

Supplementary References

1. Pertea M, Pertea GM, Antonescu CM, Chang TC, Mendell JT, Salzberg SL. StringTie enables improved reconstruction of a transcriptome from RNA-seq reads. Nat Biotechnol. 2015; 33: 290-5.

2. Langmead B, Trapnell C, Pop M, Salzberg SL. Ultrafast and memory-efficient alignment of short DNA sequences to the human genome. Genome Biol. 2009; 10: R25.

3. Trapnell C, Roberts A, Goff L, Pertea G, Kim D, Kelley DR, et al. Differential gene and transcript expression analysis of RNA-seq experiments with TopHat and Cufflinks. Nat Protoc. 2012; 7: 562-78.

4. Wang L, Feng Z, Wang X, Wang X, Zhang X. DEGseq: an R package for identifying differentially expressed genes from RNA-seq data. Bioinformatics. 2010; 26: 136-8.

5. Yang F, Zhang L, Huo XS, Yuan JH, Xu D, Yuan SX, et al. Long noncoding RNA high expression in hepatocellular carcinoma facilitates tumor growth through enhancer of zeste homolog 2 in humans. Hepatology. 2011; 54: 1679-89.

Supplementary Figures and Figure Legends

Figure S1

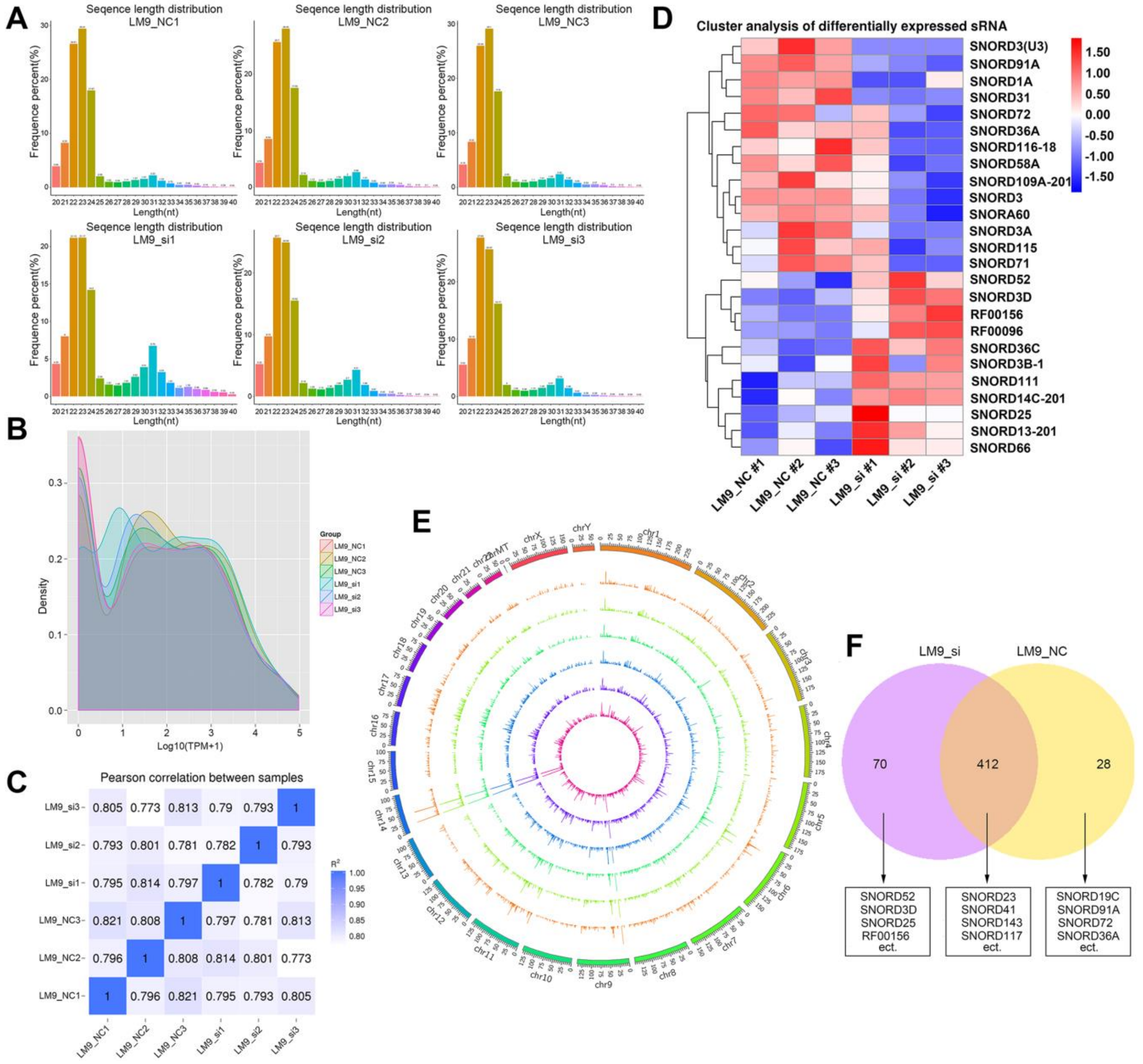

Figure S1. Bioinformatics analysis of snoRNA sequencing results. (A) The clean reads of each sample were screened for snoRNAs in a certain length range for subsequent analysis. The length distributions of snoRNAs were calculated. **(B)** Distribution diagram of snoRNA expression level TPM density. The TPM density distribution can examine the gene expression pattern of the sample as a whole. **(C)** The correlation of gene expression level between samples is an important index to test the reliability of experiment and the rationality of sample selection. The closer the correlation coefficient is to 1, the higher the similarity of expression patterns between samples. If there is biological duplication in the sample, the correlation coefficient between the biological duplication is usually higher. **(D)** Clustering analysis of differential snoRNAs. Clustering analysis of differential snoRNAs was used to judge the clustering mode of differential snoRNA expression under different experimental conditions. **(E)** Visualization results of different snoRNA genomes. **(F)** Venn diagram of different snoRNAs.

Figure S2

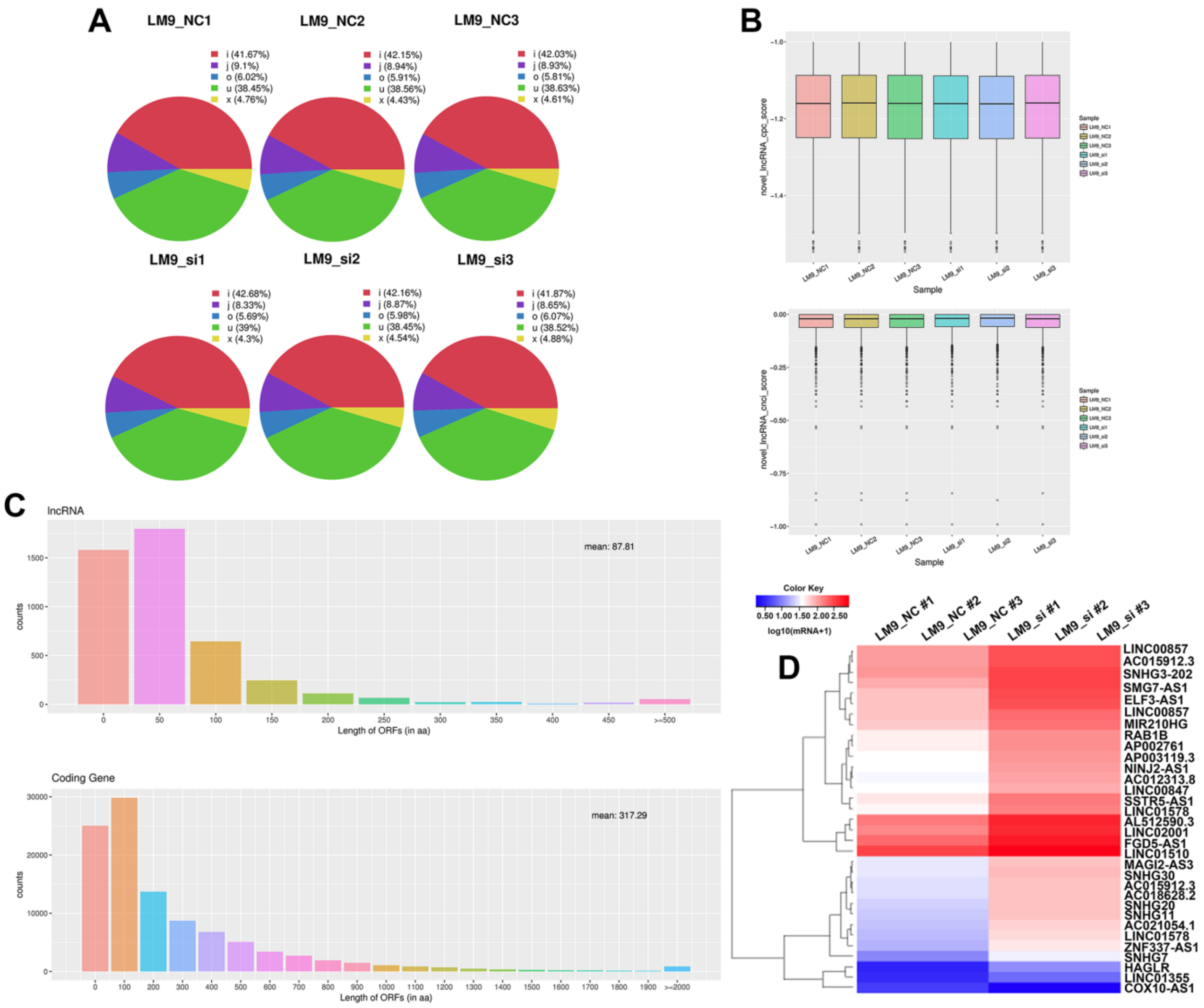

Figure S2. Bioinformatics analysis of lncRNA sequencing results. (A) The proportion of lncRNAs in each sample. J, potentially novel isoform (fragment): at least one splice junction is shared with a reference transcript; I, a transfrag falling entirely within a reference intron; O, generic exonic overlap with a reference transcript; U, unknown intergenic transcript; X, exonic overlap with reference on the opposite strand. **(B)** Box chart of score statistics of lncRNA CNCI and CPC in each sample. **(C)** The ORF distribution of lncRNAs and mRNAs. **(D)** Cluster analysis of different gene expression levels.

Figure S3

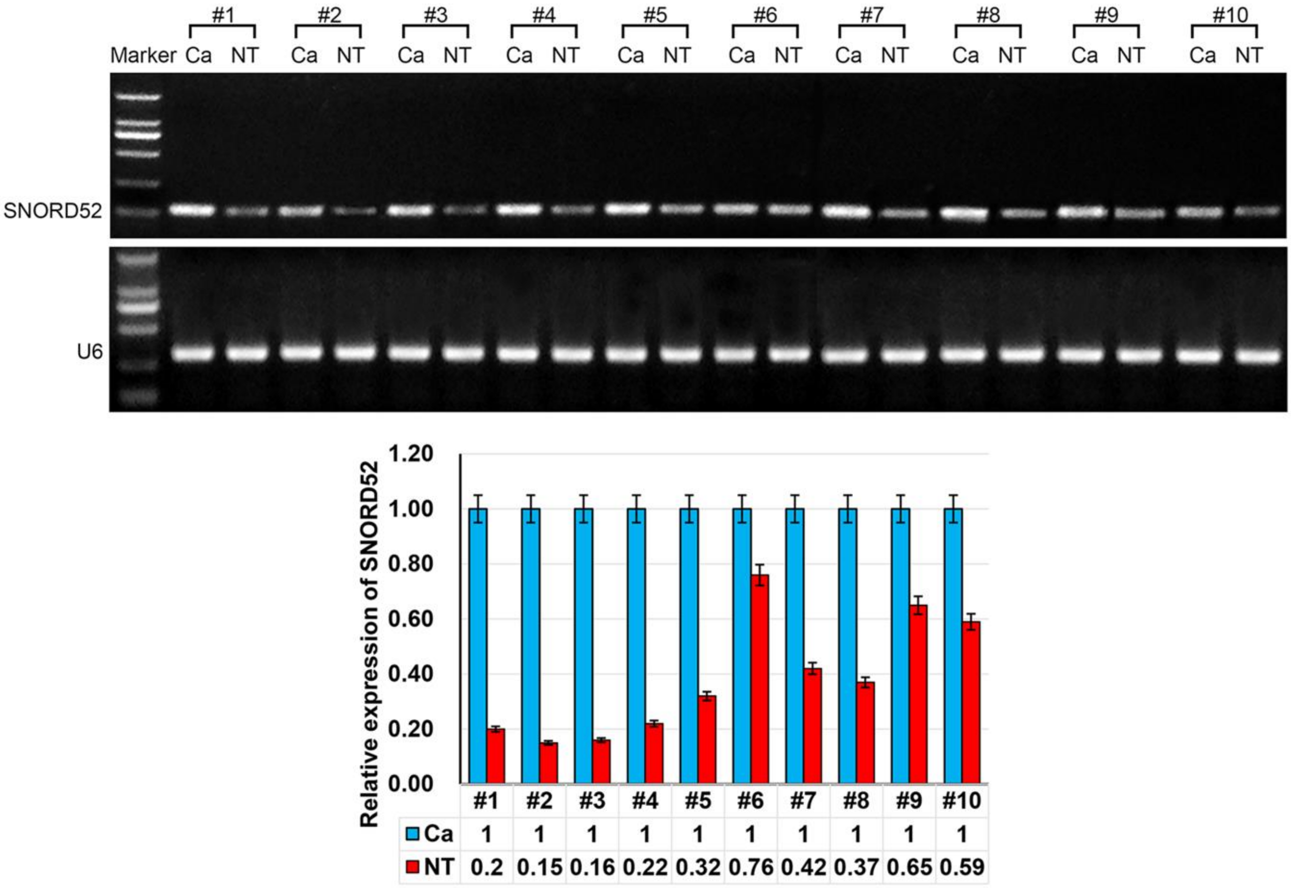

Figure S3. RT-PCR results of SNORD52 expression levels in 10 pairs of HCC tissues and adjacent tissues.

Figure S4

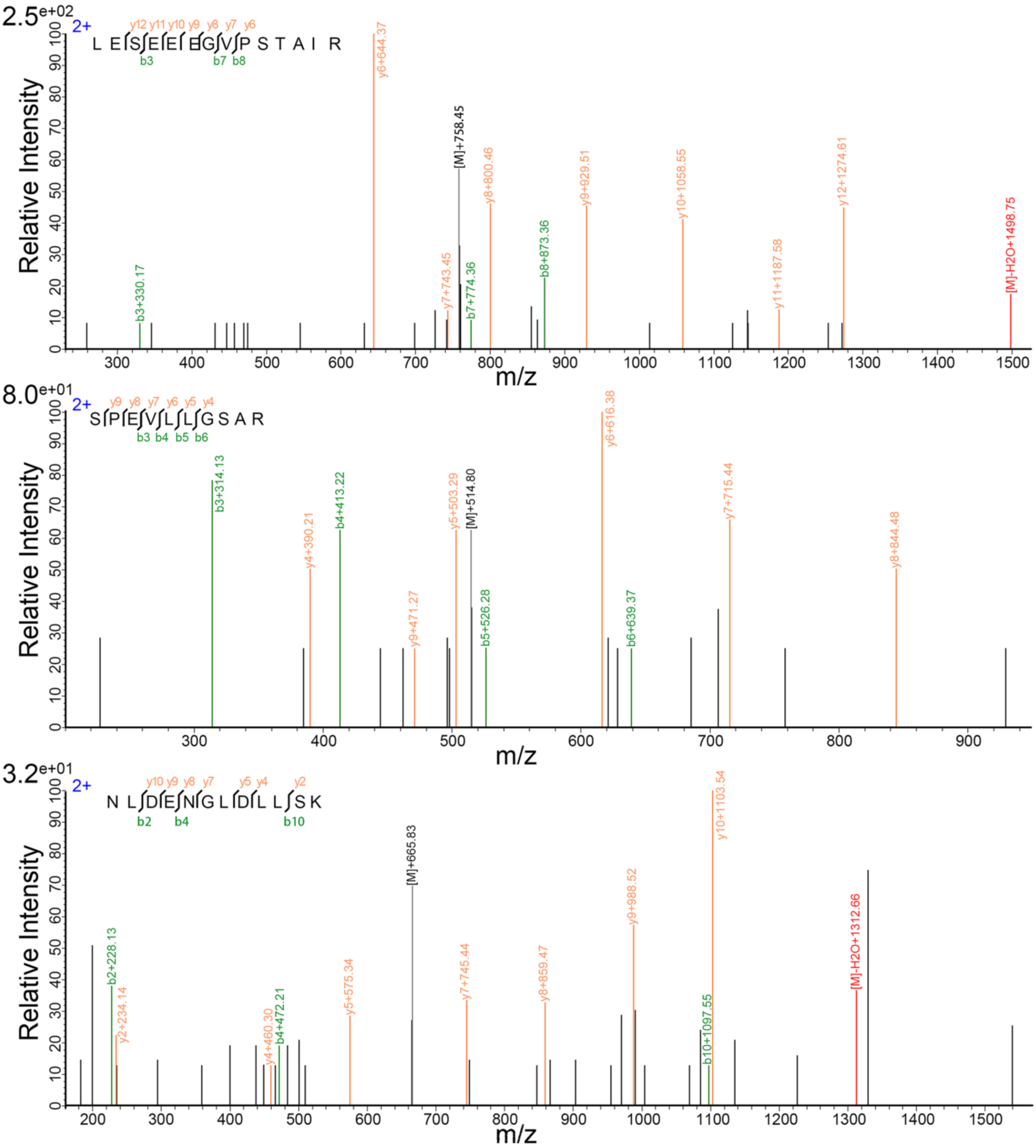

Figure S4. Mass spectrometry of CDK1 from the RNA pull-down assay.

Figure S5

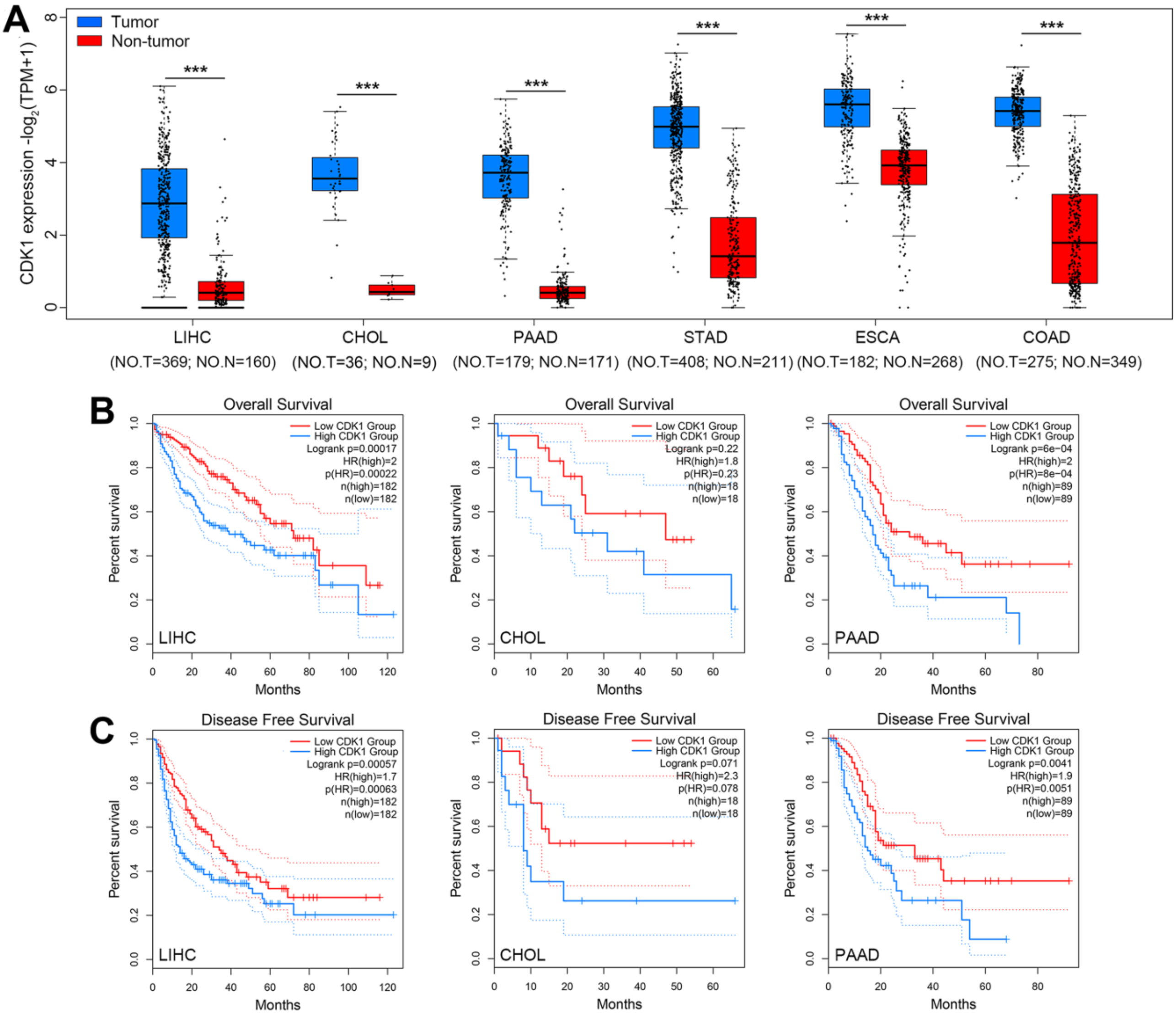

Figure S5. CDK1 was upregulated in digestive system tumors and associated with poor prognosis in hepatobiliary and pancreatic tumors. The data are from the TCGA database. (A) Quantitative RT-PCR analysis of CDK1 expression levels in liver hepatocellular carcinoma (LIHC), cholangiocarcinoma (CHOL), pancreatic adenocarcinoma (PAAD), stomach adenocarcinoma (STAD), esophageal carcinoma (ESCA) and colon adenocarcinoma (COAD). ***p<0.001. **(B-C)** Kaplan-Meier analysis of overall survival and recurrence-free survival based on CDK1 expression levels in patients with LHCC, CHOL and PAAD.

Figure S6

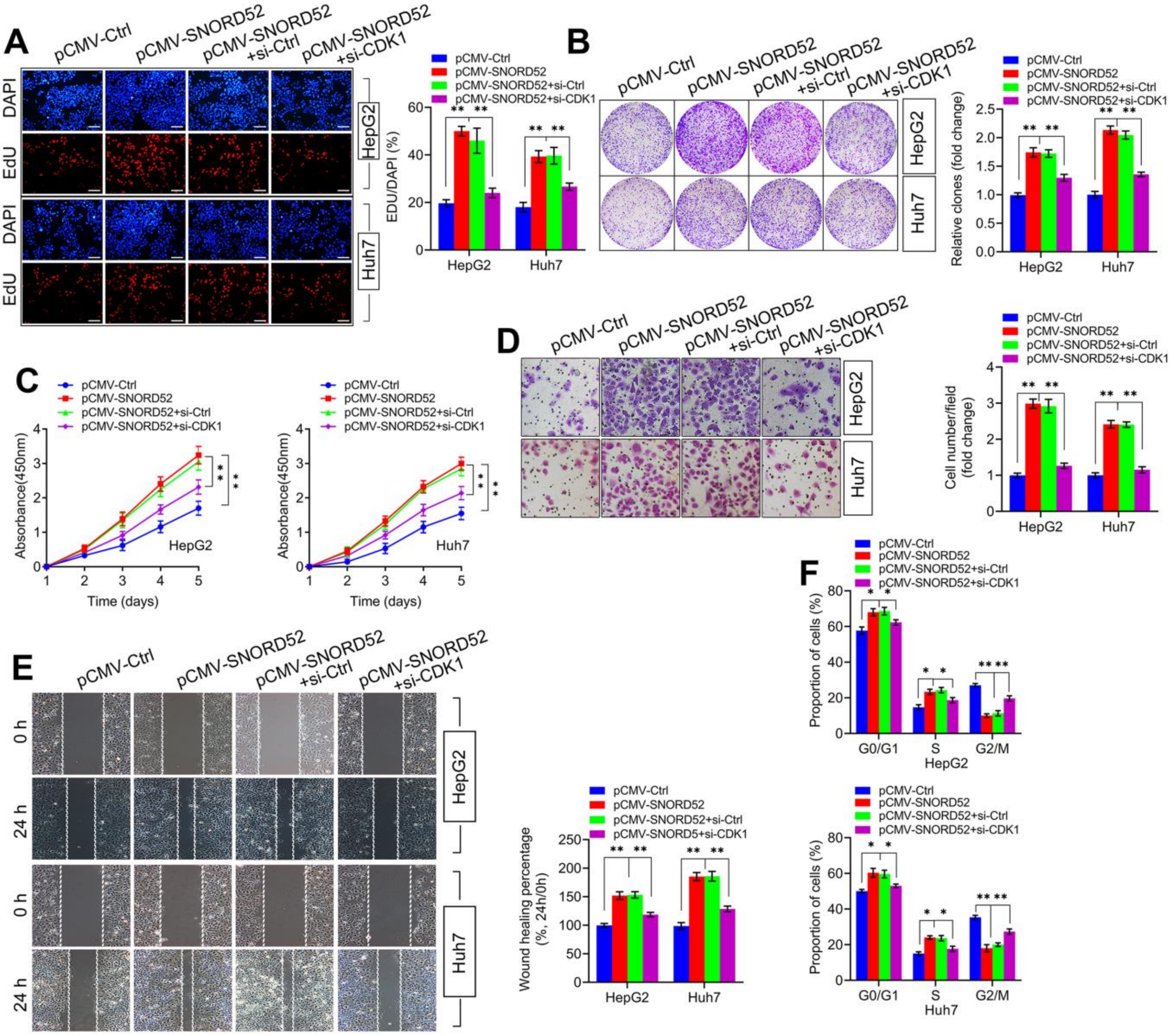

Figure S6. The biological function of SNORD52 was dependent on the presence of CDK1. si-CDK1 and control siRNA were transfected into SNORD52 overexpressed HepG2 and Huh7 cells. **(A)** The cell proliferation rate was assessed using EdU assays. *p<0.05, **p<0.01. **(B)** Colony formation assays were conducted to evaluate the proliferation ability of SNORD52-overexpressed HepG2 and Huh7 cells when si-CDK1 and control siRNA were transfected. *p<0.05, **p<0.01. **(C)** CCK-8 assays showed that the downregulation of CDK1 weakened the effect of SNORD52 on HepG2 and Huh7 cell proliferation. *p<0.05, **p<0.01. **(D)** Transwell assays showed that the downregulation of CDK1 weakened the effect of SNORD52 on HepG2 and Huh7 cell invasion. *p<0.05, **p<0.01. **(E)** Wound healing assays showed that the downregulation of CDK1 weakened the effect of SNORD52 on HepG2 and Huh7 cell migration. *p<0.05, **p<0.01. **(F)** Downregulation of CDK1 weakened the effect of SNORD52 on HepG2 and Huh7 cell cycle progression. *p<0.05, **p<0.01.

Figure S7

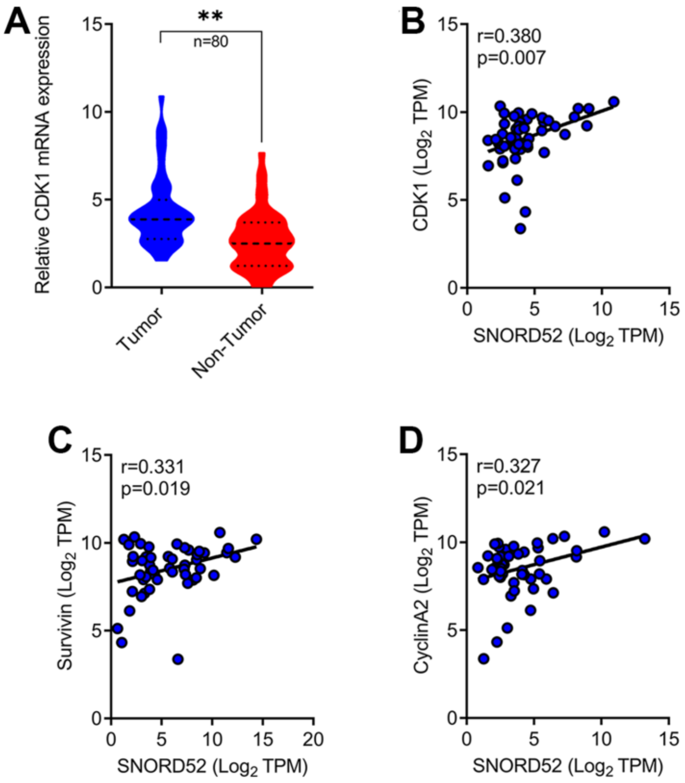

Figure S7. SNORD52 expression level was associated with high levels of CDK1 and checkpoint proteins of the G2/M phase. (A) Quantitative RT-PCR analysis of CDK1 expression in 80 patients with HCC. **$p<0.01$. **(B-D)** Bivariate correlation analysis of the relationship between SNORD52 and CDK1, Survivin, CyclinA2 expression levels in HCC tissues.

Supplementary Tables

Table S1. Primer sequence and target sequence used in this study

Target Gene	Sequence or Target Sequence
Upf1-F	5'-CTGCAACGGACGTGGAAATAC-3'
Upf1-R	5'-ACAGCCGCAGTTGTAGCAC-3'
β-actin-F	5'-AGCGAGCATCCCCCAAAGTT-3'
β-actin-R	5'-GGGCACGAAGGCTCATCATT-3'
GAPDH-F	5'-GTCTCCTCTGACTTCAACAGCG-3'
GAPDH-R	5'-ACCACCCTGTTGCTGTAGCCAA-3'
U6-F	5'-CGCTTCGGCAGCACATATAC-3'
U6-R	5'-AAATATGGAACGCTTCACGA-3'
SNORD52-F	5'-GGGAATGATGATTTCACAGACT-3'
SNORD52-R	5'-TGACATCATGACCAGCATCG-3'
SNORD3D-F	5'-CGTGTAGAGCACCGAAAACCC-3'
SNORD3D-R	5'-GCGTTCTCTCCCTCTCACTCCC-3'
RF00156-F	5'-CTGAGTTCCTCGCCTCAT-3'
RF00156-R	5'-CCTTAGAGCAGCCATACAAG-3'
RF00096-F	5'-ATCCTTACCTGTTCCTCGTT-3'
RF00096-R	5'-AGGCAGAGGCATTAATCACA-3'
CDK1-F	5'-AAACTACAGGTCAAGTGGTAGCC-3'
CDK1-R	5'-TCCTGCATAAGCACATCCTGA-3'
Survivin-F	5'-AGGACCACCGCATCTCTACAT-3'
Survivin-R	5'-AAGTCTGGCTCGTTCTCAGTG-3'
CyclinA2-F	5'-CGCTGGCGGTACTGAAGTC-3'
CyclinA2-R	5'-GAGGAACGGTGACATGCTCAT-3'
pCMV-CDK1-F	5'-CGCAAATGGGCGGTAGGCGTG-3'
pCMV-CDK1-R	5'-TAGTCAGCCATGGGGCGGAGA-3'
Upf1-siRNA #1	5'-GCGAGAAGGACUUCAUCAUTT-3'<br>5'-AUGAUGAAGUCCUUCUCGCTT-3'
Upf1-siRNA #2	5'-GCAGCCACAUUGUAAAUCATT-3'<br>5'-UGAUUUACAAUGUGGCUGCTT-3'
Upf1-siRNA #3	5'-CCUACCAGUACCAGAACAUTT-3'<br>5'-AUGUUCUGGUACUGGUAGGTT-3'
Control-siRNA	5'-UUCUCCGAACGUGUCACGUTT-3'<br>5'-ACGUGACACGUUCGGAGAATT-3’
CDK1-siRNA	5'-UACAGUACUGUGAUAACUGAA-3'<br>5'-CAGUUAUCACAGUACUGUAUU-3'
Control ASO	5'- mUmCmAmCmCTTCACCCTCTmCmCmAmCmU-3'
SNORD52 ASO	5'- mGmGmAmUmUATCCCACCTGmAmCmGmAmU-3'
SNORD52 Sense-F	5'-TAATACGACTCACTATAGGGGGGAATGATGATTTCACAGACTAG-3'
SNORD52 Sense-R	5'-GAGTCAGAACTTAGTTTTGACATC-3'
SNORD52 Antisense-F	5'-TAATACGACTCACTATAGGGGAGTCAGAACTTAGTTTTGACATC-3'
SNORD52 Antisense-R	5'-GGGAATGATGATTTCACAGACTAG-3'

Table S2. Antibody information used in this study

Antibody	WB	IHC	IF	Specificity	Company
Upf1(#12040S)	1:1000			Rabbit polyclona	Cell Signaling Technology
GAPDH(KM9002)	1:5000			Mouse monoclonal	Sungene Biotechnology
β-actin(66009-1-lg)	1:5000			Mouse monoclonal	Proteintech
Survivin(ab76424)	1:5000	1:500		Rabbit polyclona	Abcam
p53(ab131442)	1:5000			Rabbit polyclonal	Abcam
p-p53(#2521)	1:1000			Rabbit polyclona	Cell Signaling Technology
CyclinA2(ab181591)	1:5000	1:500		Mouse monoclonal	Abcam
CDK1(ab133327)	1:5000	1:500	1:200	Rabbit monoclonal	Abcam
p-CDK1(#4539)	1:1000			Rabbit polyclona	Cell Signaling Technology
CyclinB1(#12231)	1:1000		1:100	Rabbit polyclona	Cell Signaling Technology
Ki-67(sc-15402)		1:100		Rabbit polyclona	Santa Cruz Biotechnology

Table S3. The list of top deregulated snoRNAs from RNA-seq

snoRNAs_name	LM9_si_readcount	LM9_NC_readcount	Log2FoldChange	P val	P adj
ENST00000583541.1 (SNORD52)	111.2731564	10.44783119	1.663	0.000755	0.055132
ENST00000630092.1 (SNORD3D)	128.5635842	33.30184316	1.5816	3.41E-05	0.004982
ENST00000365075.1 (RF00156)	6.268963647	0	1.4056	0.011562	NA
ENST00000363156.1 (RF00096)	28.74808645	4.392867195	1.3565	0.005938	NA
ENST00000384289.1	28.90790862	7.459107062	1.1684	0.015158	NA
ENST00000617128.1	979.3568219	195.9754103	1.1578	0.00348	0.10161
ENST00000363485.1	2.953625533	0	0.98557	0.028883	NA
ENST00000408139.1	49.0889136	23.93056114	0.86465	0.013021	NA
ENST00000365382.1	98.68457539	53.30554917	0.82384	0.002048	0.099657
ENST00000365607.2	1588.441543	863.1393363	0.74989	0.032084	0.42584
ENST00000459299.1	725.2286128	387.3849153	0.74924	0.043505	0.44339
ENST00000390856.1	510.996204	289.8634238	0.69659	0.045572	0.44339
ENST00000364805.1	30.40130523	17.45075125	0.678	0.048936	NA
ENST00000386745.1	143.2937884	96.87706591	0.51737	0.036903	0.44339
ENST00000384048.1	787.8541806	591.4769367	0.40314	0.011191	0.20423
ENST00000410413.1	896.0234232	1154.509775	-0.35309	0.048591	0.44339
ENST00000390861.1	150.1460736	232.5471632	-0.59086	0.028118	0.41052
ENST00000364968.1	198.1942133	340.6301926	-0.6706	0.047501	0.44339
ENST00000362607.1	36.64799719	71.72780816	-0.84663	0.005262	0.12804
ENST00000390994.1	84.79991191	169.0761283	-0.89945	0.003109	0.10161
ENST00000384221.1	17.23689317	48.19303218	-0.9384	0.049717	NA
ENST00000383961.1	2.597221022	12.6133019	-0.98843	0.045272	NA
ENST00000383875.1	256.551286	641.623338	-1.0012	0.01769	0.28697
ENST00000459128.1	7.340556133	21.27475918	-1.0254	0.027202	NA
ENST00000384034.2	5.240708466	18.42852447	-1.1576	0.015229	NA
ENST00000362396.1	0.473378614	4.476734564	-1.2313	0.011128	NA
ENST00000620232.1	245.9763942	1773.62952	-1.2511	0.0111	0.20423

Table S4. The list of top deregulated lncRNAs from RNA-seq

LncRNA_name	Length	Gene_name	Log2(fc)	P val	Regulation	Significant
ENST00000451424	2161	LINC00857	11.85	0.00	up	yes
ENST00000566551	348	AC015912.3	10.95	0.00	up	yes
ENST00000437681	3361	AP002761.4	10.86	0.00	up	yes
ENST00000624060	1112	AC087388.1	10.70	0.00	up	yes
ENST00000419190	788	ELF3-AS1	10.70	0.00	up	yes
ENST00000422847	4382	AC011815.1	10.54	0.00	up	yes
ENST00000500447	3819	AC018628.1	10.46	0.00	up	yes
ENST00000526951	2538	AL137003.2	10.36	0.00	up	yes
ENST00000565433	8149	Z82217.1	10.14	0.00	up	yes
ENST00000566747	2437	AC005154.1	9.97	0.00	up	yes
ENST00000537514	2005	AC005332.7	9.81	0.00	up	yes
ENST00000550601	5402	AC012313.8	9.17	0.00	up	yes
ENST00000617013	1809	LINC00847	7.37	0.00	up	yes
ENST00000556895	2864	SSTR5-AS1	6.35	0.01	up	yes
ENST00000569832	6269	AL512590.3	5.04	0.00	up	yes
ENST00000563777	1504	LINC02001	4.88	0.00	up	yes
ENST00000571370	3792	FGD5-AS1	4.08	0.02	up	yes
ENST00000623598	468	LINC01510	3.93	0.04	up	yes
ENST00000573866	8524	MAGI2-AS3	3.55	0.02	up	yes
ENST00000638682	2471	AL136537.2	3.37	0.03	up	yes
ENST00000619432	637	AC007952.4	3.23	0.04	up	yes
ENST00000624147	453	AP001107.2	3.05	0.02	up	yes
ENST00000614046	2143	SNHG20	2.89	0.02	up	yes
ENST00000434411	1058	SNHG11	2.60	0.03	up	yes
ENST00000586922	2338	AC021054.1	2.55	0.03	up	yes
ENST00000616428	802	LINC01578	2.45	0.04	up	yes
ENST00000539278	4666	ZNF337-AS1	2.21	0.05	up	yes
ENST00000598112	789	SNHG7	2.13	0.01	up	yes
ENST00000623179	2614	SNHG3	-3.17	0.01	down	yes
ENST00000595059	2195	CU634019.1	-3.71	0.03	down	yes
ENST00000448494	2351	LINC00342	-3.87	0.05	down	yes
ENST00000416928	4443	PINK1-AS	-4.86	0.00	down	yes
ENST00000414393	4235	HAGLR	-4.97	0.01	down	yes
ENST00000434729	2246	SMG7-AS1	-5.37	0.03	down	yes
ENST00000615804	1965	MIR210HG	-6.90	0.01	down	yes
ENST00000625157	2198	AC005229.4	-9.64	0.00	down	yes
ENST00000424349	4739	AC010336.1	-9.93	0.00	down	yes
ENST00000562049	6721	AC110048.2	-10.28	0.00	down	yes
ENST00000610159	1473	AC024075.2	-10.31	0.00	down	yes
ENST00000501937	5444	LINC01355	-10.47	0.00	down	yes
ENST00000606924	711	AC068888.1	-10.50	0.00	down	yes
ENST00000584621	2072	AC068473.5	-10.53	0.00	down	yes

lncRNA_name	length	gene_name	log2(fc)	pval	regulation	significant
ENST00000452320	5334	AC097376.2	-10.75	0.00	down	yes
ENST00000441991	1917	COX10-AS1	-11.82	0.00	down	yes
ENST00000610085	2995	AC007191.1	-11.86	0.00	down	yes
ENST00000375206	4192	AP003119.3	-12.07	0.00	down	yes
ENST00000416970	3951	FBXL19-AS1	-12.08	0.00	down	yes

Table S5. The correlation between SNORD52 expression and clinicopathological features in 80 HCC patients

Characteristics	No. of cases (%)	Low expression n=40	High expression n=40	P value
**Age**				0.25
≥58	51 (63.75%)	28 (70.00%)	23 (57.50%)	
＜58	29 (36.25%)	12 (30.00%)	17 (42.50%)	
**Gender**				0.56
Male	66 (82.50%)	34 (85.00%)	32 (80.00%)	
Female	14 (17.50%)	6 (15.00%)	8 (20.00%)	
**Serum AFP**				0.14
≥400μg/L	56 (70.00%)	31 (77.50%)	25 (62.50%)	
＜400μg/L	24 (30.00%)	9 (22.50%)	15 (37.50%)	
**Tumor Size**				0.64
≥5cm	52 (65.00%)	27 (67.50%)	25 (62.50%)	
＜5cm	28 (35.00%)	13 (32.50%)	15 (37.50%)	
**TNM Stage**				**<0.01*** **
I-II	57 (71.25%)	35 (87.50%)	22 (55.00%)	
III-IV	23 (28.75%)	5 (12.50%)	18 (45.00%)	
**HBV Infection**				0.41
Present	63 (78.75%)	33 (82.50%)	30 (75.00%)	
Absent	17 (21.25%)	7 (17.50%)	10 (25.00%)	
**Liver Cirrhosis**				0.11
Present	47 (58.75%)	20 (50.00%)	27 (67.50%)	
Absent	33 (41.25%)	20 (50.00%)	13 (32.50%)	
**Microvascular Invasion**				**<0.01*** **
Present	21 (26.25%)	3 (7.50%)	18 (45.00%)	
Absent	59 (73.75%)	37 (92.50%)	22 (55.00%)	

Table S6. The proteins identified by mass spectrometry analysis

No.	Unused	Acc	Coverage (%)	Length	Mass	#Unique Peptide	#Unique Spectrum
1	34.77	sp|P11142|HSP7C_HUMAN	26.62999928	646	70897.6	16	31
2	31.12	sp|P08238|HS90B_HUMAN	21.26999944	724	83263.5	12	20
4	30.74	sp|Q13085|ACACA_HUMAN	7.927999645	2346	265551.7	14	26
5	29.08	sp|P60709|ACTB_HUMAN	41.60000086	375	41736.4	2	9
6	27.93	sp|Q08211|DHX9_HUMAN	12.43999973	1270	140957.5	12	21
7	27.9	sp|P07437|TBB5_HUMAN	41.22000039	444	49670.5	4	7
8	27.41	sp|Q7KZF4|SND1_HUMAN	18.12999994	910	101996.1	12	19
9	26.49	sp|P04406|G3P_HUMAN	36.4199996	335	36053	14	40
10	22.45	sp|O43707|ACTN4_HUMAN	16.47000015	911	104853.2	11	21
11	21.76	sp|P11021|BIP_HUMAN	25.83999932	654	72332.4	12	21
12	19.33	sp|P13639|EF2_HUMAN	14.45000023	858	95337.4	11	21
14	18.08	sp|P25705|ATPA_HUMAN	17.54000038	553	59750.1	8	12
15	17.95	sp|Q71U36|TBA1A_HUMAN	23.72999936	451	50135.2	5	8
16	17.52	sp|P07900|HS90A_HUMAN	17.75999963	732	84659	10	16
17	17.29	sp|P38646|GRP75_HUMAN	14.7300005	679	73680	8	15
18	17.26	sp|P11498|PYC_HUMAN	7.554999739	1178	129632.6	8	15
19	16.18	sp|P06576|ATPB_HUMAN	17.96000004	529	56559.4	8	11
20	16.08	sp|P14618|KPYM_HUMAN	20.53000033	531	57936.4	8	17
22	15.84	sp|P16403|H12_HUMAN	23.46999943	213	21364.6	3	5
23	15.68	sp|P02545|LMNA_HUMAN	15.65999985	664	74138.8	8	11
24	14.83	sp|P08195|4F2_HUMAN	13.49000037	630	67993.3	7	11
25	14.76	sp|Q96AE4|FUBP1_HUMAN	13.19999993	644	67560.2	7	12
26	14.74	sp|P04075|ALDOA_HUMAN	21.15000039	364	39419.7	8	10
27	14.49	sp|P06733|ENOA_HUMAN	24.65000004	434	47168.6	8	17
28	14.34	sp|P07195|LDHB_HUMAN	24.85000044	334	36638.2	5	5
29	13.72	sp|P04843|RPN1_HUMAN	12.69000024	607	68568.8	7	9
30	13.69	sp|P07355|ANXA2_HUMAN	23.60000014	339	38603.6	7	10
31	12.84	sp|P30101|PDIA3_HUMAN	19.21000034	505	56781.8	7	13
32	12.68	sp|P07237|PDIA1_HUMAN	10.23999974	508	57115.8	6	8
33	12.55	sp|P22314|UBA1_HUMAN	7.089000195	1058	117848.1	6	14
34	12.28	sp|P31327|CPSM_HUMAN	5.133000016	1500	164938.1	6	9
35	12.15	sp|P05023|AT1A1_HUMAN	8.211000264	1023	112895	7	11
36	11.6	sp|P08670|VIME_HUMAN	14.38000053	466	53651.2	6	7
37	10.66	sp|P10809|CH60_HUMAN	12.56999969	573	61054.2	5	13
38	10.63	sp|Q00839|HNRPU_HUMAN	8.606000245	825	90584.1	5	11
39	10.19	sp|P62826|RAN_HUMAN	22.68999964	216	24423	4	7
40	10	sp|P26641|EF1G_HUMAN	16.48000032	437	50118.4	5	7
41	10	sp|Q8N163|CCAR2_HUMAN	8.991999924	923	102900.6	5	7

No.	Unused	Acc	Coverage (%)	Length	Mass	#Unique Peptide	#Unique Spectrum
43	9.91	sp|P00338|LDHA_HUMAN	17.47000068	332	36688.5	5	7
44	9.46	sp|Q15393|SF3B3_HUMAN	5.834000185	1217	135576.2	5	7
45	9.12	sp|P26038|MOES_HUMAN	9.358999878	577	67819.6	2	2
46	9.06	sp|P50990|TCPQ_HUMAN	9.488999844	548	59620.1	4	8
48	8.95	sp|P05783|K1C18_HUMAN	14.88000005	430	48057.4	5	9
49	8.81	sp|P17987|TCPA_HUMAN	7.914000005	556	60342.9	4	7
50	8.7	sp|P67809|YBOX1_HUMAN	29.01000082	324	35924.1	5	5
51	8.67	sp|P00966|ASSY_HUMAN	10.67999974	412	46530.1	4	5
52	8.67	sp|P62937|PPIA_HUMAN	38.78999949	165	18012.4	5	13
53	8.66	sp|Q92945|FUBP2_HUMAN	9.564000368	711	73115.2	4	6
56	8.45	sp|P04083|ANXA1_HUMAN	13.86999935	346	38713.9	4	4
57	8.28	sp|P40926|MDHM_HUMAN	14.79000002	338	35502.9	4	9
58	8.23	sp|Q15366|PCBP2_HUMAN	16.70999974	365	38579.7	2	4
59	8.18	sp|P02786|TFR1_HUMAN	7.631999999	760	84870.7	4	9
60	8.12	sp|P29401|TKT_HUMAN	10.75000018	623	67876.9	4	9
61	8.09	sp|P04792|HSPB1_HUMAN	32.67999887	205	22782.3	5	11
62	8	sp|P60842|IF4A1_HUMAN	13.30000013	406	46153.5	4	6
63	8	sp|P08865|RSSA_HUMAN	16.60999954	295	32853.8	4	5
64	7.96	sp|P80723|BASP1_HUMAN	51.09999776	227	22693.2	4	4
65	7.9	sp|P00558|PGK1_HUMAN	17.50999987	417	44614.4	5	10
66	7.74	sp|P21333|FLNA_HUMAN	2.342000045	2647	280737.6	4	5
67	7.7	sp|P00367|DHE3_HUMAN	10.04000008	558	61397.3	4	6
68	7.66	sp|P14625|ENPL_HUMAN	5.728999898	803	92468.1	4	8
69	7.57	sp|P11586|C1TC_HUMAN	5.775000155	935	101558.4	4	5
70	7.49	sp|P60174|TPIS_HUMAN	19.58000064	286	30790.8	4	5
71	7.43	sp|P13489|RINI_HUMAN	11.50000021	461	49972.8	4	4
72	7.42	sp|O95573|ACSL3_HUMAN	7.917000353	720	80419.4	4	5
73	7.32	sp|P09211|GSTP1_HUMAN	19.51999962	210	23355.6	4	15
74	7.3	sp|P22626|ROA2_HUMAN	16.70999974	353	37429.7	4	8
75	7.21	sp|P55072|TERA_HUMAN	6.82400018	806	89320.9	4	7
76	7.11	sp|P61978|HNRPK_HUMAN	8.639000356	463	50975.8	4	7
77	6.94	sp|Q13838|DX39B_HUMAN	7.242999971	428	48990.9	3	3
78	6.85	sp|P52272|HNRPM_HUMAN	3.973000124	730	77515.3	3	5
79	6.76	sp|P06493|CDK1_HUMAN	12.12000027	297	34095.1	3	4
80	6.57	sp|P40939|ECHA_HUMAN	6.029000133	763	82999	3	4
81	6.36	sp|Q06210|GFPT1_HUMAN	5.722000077	699	78805.8	3	4
82	6.34	sp|P55060|XPO2_HUMAN	2.883999981	971	110415.4	2	4
83	6.29	sp|Q06830|PRDX1_HUMAN	16.58000052	199	22110.2	2	4
84	6.23	sp|Q9H2U1|DHX36_HUMAN	3.869000077	1008	114759.3	3	4
85	6.2	sp|P34932|HSP74_HUMAN	4.881000146	840	94330.2	2	3

No.	Unused	Acc	Coverage (%)	Length	Mass	#Unique Peptide	#Unique Spectrum
87	6.15	sp|P41091|IF2G_HUMAN	10.17000005	472	51109.1	3	6
88	6.12	sp|Q9NR30|DDX21_HUMAN	3.830999881	783	87343.9	3	3
89	6.11	sp|P22102|PUR2_HUMAN	4.554000124	1010	107766.3	3	5
91	6.07	sp|O43390|HNRPR_HUMAN	4.106999934	633	70942.8	3	4
92	6.01	sp|Q9BW7|SFXN3_HUMAN	13.40000033	321	35503.1	3	4
93	6.01	sp|P31943|HNRH1_HUMAN	10.23999974	449	49229.2	3	5
94	6	sp|Q16555|DPYL2_HUMAN	9.091000259	572	62293.1	3	3
95	6	sp|Q14498|RBM39_HUMAN	8.112999797	530	59379	3	4
96	6	sp|P21796|VDAC1_HUMAN	13.77999932	283	30772.4	3	6
97	6	sp|P62888|RL30_HUMAN	40.86999893	115	12783.9	3	6
98	5.91	sp|Q99832|TCPH_HUMAN	6.998000294	543	59366.1	3	4
99	5.87	sp|Q13283|G3BP1_HUMAN	10.93999967	466	52164	3	5
100	5.86	sp|P27797|CALR_HUMAN	8.152999729	417	48141.2	3	3
101	5.74	sp|Q13423|NNTM_HUMAN	4.143999889	1086	113894.6	3	4
102	5.72	sp|P50991|TCPD_HUMAN	6.30799979	539	57923.6	3	4
103	5.71	sp|P62805|H4_HUMAN	29.12999988	103	11367.3	3	4
104	5.7	sp|P12004|PCNA_HUMAN	15.71000069	261	28768.5	3	5
105	5.66	sp|Q00325|MPCP_HUMAN	5.248999968	362	40094.5	3	5
106	5.63	sp|P53985|MOT1_HUMAN	9.399999678	500	53943.7	3	3
107	5.58	sp|P16401|H15_HUMAN	15.03999978	226	22579.9	3	3
108	5.58	sp|P37837|TALDO_HUMAN	10.39000005	337	37539.7	3	3
109	5.46	sp|P25786|PSA1_HUMAN	18.62999946	263	29555.3	3	3
110	5.45	sp|P53621|COPA_HUMAN	3.430999815	1224	138344.6	3	3
111	5.41	sp|P05141|ADT2_HUMAN	11.41000018	298	32852	2	4
113	5.25	sp|P49411|EFTU_HUMAN	9.291999787	452	49541.1	3	4
114	5.2	sp|P00505|AATM_HUMAN	6.278999895	430	47517.3	2	2
115	5.19	sp|Q12906|ILF3_HUMAN	2.907999977	894	95338	3	3
116	5.14	sp|Q9UJS0|CMC2_HUMAN	5.629999936	675	74175	3	4
117	5.1	sp|Q00610|CLH1_HUMAN	2.089999989	1675	191613	3	4
118	5.04	sp|Q12931|TRAP1_HUMAN	7.385999709	704	80109.2	4	7
119	5.02	sp|P18124|RL7_HUMAN	16.94000065	248	29225.6	3	5
120	4.85	sp|P26640|SYVC_HUMAN	2.452999912	1264	140474.8	2	3
121	4.8	sp|P05166|PCCB_HUMAN	5.380000174	539	58215.1	2	3
122	4.66	sp|P27695|APEX1_HUMAN	12.25999966	318	35554.2	2	2
123	4.65	sp|P23396|RS3_HUMAN	17.28000045	243	26688.1	3	5
125	4.57	sp|P22087|FBRL_HUMAN	8.100000024	321	33784.1	2	2
126	4.52	sp|P39656|OST48_HUMAN	4.385999963	456	50800.3	2	5
127	4.45	sp|Q07020|RL18_HUMAN	12.7700001	188	21634.3	2	2
128	4.41	sp|P13667|PDIA4_HUMAN	4.96100001	645	72931.9	3	2

No.	Unused	Acc	Coverage (%)	Length	Mass	#Unique Peptide	#Unique Spectrum
129	4.37	sp|P22392|NDKB_HUMAN	13.81999999	152	17297.9	2	3
130	4.36	sp|P62979|RS27A_HUMAN	18.59000027	156	17964.8	2	4
131	4.33	sp|P49588|SYAC_HUMAN	2.583000064	968	106809.5	2	2
132	4.31	sp|P23284|PPIB_HUMAN	12.03999966	216	23742.4	2	7
133	4.25	sp|P22695|QCR2_HUMAN	9.272000194	453	48442.6	3	5
134	4.22	sp|Q07065|CKAP4_HUMAN	7.309000194	602	66022	3	3
135	4.18	sp|P19338|NUCL_HUMAN	2.676000074	710	76613.9	2	4
136	4.15	sp|P07954|FUMH_HUMAN	6.274999678	510	54636.6	2	4
137	4.12	sp|P51149|RAB7A_HUMAN	13.0400002	207	23489.5	2	2
139	4.09	sp|P24539|AT5F1_HUMAN	10.15999988	256	28908.5	3	3
140	4.07	sp|P35232|PHB_HUMAN	8.088000119	272	29803.8	2	4
141	4.03	sp|P68371|TBB4B_HUMAN	40.90000093	445	49830.7	3	6
142	4.03	sp|Q14683|SMC1A_HUMAN	3.162999824	1233	143231.9	2	2
143	4.03	sp|P62258|1433E_HUMAN	9.019999951	255	29173.6	2	3
144	4.01	sp|P14550|AK1A1_HUMAN	8.614999801	325	36572.7	2	3
146	4	sp|Q9BUF5|TBB6_HUMAN	21.29999995	446	49856.8	3	2
147	4	sp|Q14103|HNRPD_HUMAN	6.761000305	355	38434.1	2	3
148	4	sp|P35221|CTNA1_HUMAN	3.311000019	906	100070.3	2	2
149	4	sp|O43143|DHX15_HUMAN	3.395999968	795	90932	2	2
151	4	sp|P63104|1433Z_HUMAN	9.387999773	245	27744.8	2	2
152	4	sp|P46776|RL27A_HUMAN	8.78399983	148	16561.4	2	2
153	4	sp|Q9Y230|RUVB2_HUMAN	4.535999894	463	51156.1	2	2
154	4	sp|Q14165|MLEC_HUMAN	10.62000021	292	32233.6	2	3
155	4	sp|Q04837|SSBP_HUMAN	18.24000031	148	17259.6	2	3
156	4	sp|Q02978|M2OM_HUMAN	10.18999964	314	34061.4	2	2
157	4	sp|Q01518|CAP1_HUMAN	6.105000153	475	51901.1	2	3
158	4	sp|P61981|1433G_HUMAN	9.312000126	247	28302.3	2	4
159	4	sp|P35637|FUS_HUMAN	7.224000245	526	53426	2	2
160	4	sp|P30041|PRDX6_HUMAN	10.27000025	224	25034.7	2	3
161	3.96	sp|P62906|RL10A_HUMAN	13.35999966	217	24831.1	2	6
162	3.96	sp|P30419|NMT1_HUMAN	5.040000007	496	56805.9	2	2
163	3.89	sp|P53396|ACLY_HUMAN	2.270999923	1101	120838.3	2	2
164	3.88	sp|P31948|STIP1_HUMAN	5.525000021	543	62638.7	2	3
165	3.87	sp|P49368|TCPG_HUMAN	4.219999909	545	60533.3	2	4
166	3.82	sp|Q96EP5|DAZP1_HUMAN	7.124999911	407	43383.3	3	3
167	3.77	sp|P16615|AT2A2_HUMAN	2.686999924	1042	114755.8	2	3
168	3.76	sp|O60701|UGDH_HUMAN	5.262999982	494	55023.5	2	2
169	3.62	sp|P31939|PUR9_HUMAN	4.222999886	592	64615.3	2	2
170	3.54	sp|P26599|PTBP1_HUMAN	6.215000153	531	57220.9	2	3
171	3.52	sp|P62917|RL8_HUMAN	6.615000218	257	28024.5	2	2

No.	Unused	Acc	Coverage (%)	Length	Mass	#Unique Peptide	#Unique Spectrum
172	3.48	sp|O00299|CLIC1_HUMAN	12.4499999	241	26922.5	2	3
173	3.44	sp|P62241|RS8_HUMAN	12.5	208	24205	2	3
174	3.43	sp|P61247|RS3A_HUMAN	10.61000004	264	29944.8	2	2
175	3.39	sp|P40227|TCPZ_HUMAN	5.273000151	531	58023.6	2	2
176	3.36	sp|Q14974|IMB1_HUMAN	3.539000079	876	97169.2	2	2
177	3.34	sp|Q8WM4|PDC6I_HUMAN	3.341000155	868	96022.3	2	2
178	3.31	sp|P39023|RL3_HUMAN	5.211000144	403	46108.7	2	2
179	3.27	sp|P42704|LPPRC_HUMAN	0.860800035	1394	157903.4	1	1
181	3.21	sp|O95347|SMC2_HUMAN	2.171999961	1197	135655.1	2	2
182	3.19	sp|P48047|ATPO_HUMAN	10.32999977	213	23277.1	2	2
183	3.06	sp|P08758|ANXA5_HUMAN	2.811999992	320	35936.4	1	1
184	3.02	sp|P26373|RL13_HUMAN	9.004999697	211	24261.3	2	2
185	3	sp|P07737|PROF1_HUMAN	20.0000003	140	15054.1	2	6
186	2.98	sp|Q12905|ILF2_HUMAN	6.409999728	390	43061.8	2	2
187	2.98	sp|P51659|DHB4_HUMAN	3.804000095	736	79685.7	2	2
188	2.96	sp|P09525|ANXA4_HUMAN	6.897000223	319	35882.4	2	2
189	2.94	sp|Q9NVI7|ATD3A_HUMAN	3.784999996	634	71368.6	2	2
190	2.94	sp|P36776|LONM_HUMAN	3.127999976	959	106488.4	2	2
191	2.93	sp|P26583|HMGB2_HUMAN	13.40000033	209	24033.6	2	2
192	2.92	sp|Q92841|DDX17_HUMAN	3.291999921	729	80271.8	2	2
193	2.87	sp|O95831|AIFM1_HUMAN	1.79399997	613	66900.1	1	1
194	2.87	sp|Q9P258|RCC2_HUMAN	6.51300028	522	56084.1	2	2
195	2.86	sp|P50454|SERPH_HUMAN	5.502000079	418	46440.1	2	3
196	2.8	sp|Q8N1F7|NUP93_HUMAN	1.58699993	819	93487.4	1	1
197	2.76	sp|P62249|RS16_HUMAN	6.848999858	146	16445.2	1	1
198	2.75	sp|P19525|E2AK2_HUMAN	2.359000035	551	62093.7	1	1
199	2.74	sp|P52597|HNRPF_HUMAN	3.855000064	415	45671.6	1	1
200	2.69	sp|Q8NBI5|S43A3_HUMAN	3.054999933	491	54528.2	1	1
201	2.64	sp|Q96I24|FUBP3_HUMAN	2.448000014	572	61640.1	1	1
202	2.63	sp|P35613|BASI_HUMAN	4.156000167	385	42200.1	1	1
203	2.62	sp|P13797|PLST_HUMAN	2.063000016	630	70810.4	1	1
204	2.59	sp|P19367|HXK1_HUMAN	2.071999945	917	102485.1	2	2
205	2.59	sp|Q13247|SRSF6_HUMAN	6.685999781	344	39586.3	2	2
206	2.57	sp|P26639|SYTC_HUMAN	1.38299996	723	83434.5	1	3
207	2.5	sp|O00410|IPO5_HUMAN	2.278999984	1097	123628.9	2	2
208	2.48	sp|P46940|IQGA1_HUMAN	0.603500009	1657	189250.4	1	1
209	2.47	sp|Q96AG4|LRC59_HUMAN	3.909000009	307	34930.1	2	3
210	2.46	sp|P06744|G6PI_HUMAN	1.97100006	558	63146.7	1	1
211	2.46	sp|P31930|QCR1_HUMAN	2.500000037	480	52645.3	1	1
212	2.41	sp|O75390|CISY_HUMAN	2.360999957	466	51712	1	1

No.	Unused	Acc	Coverage (%)	Length	Mass	#Unique Peptide	#Unique Spectrum
213	2.4	sp|Q08J23|NSUN2_HUMAN	1.565000042	767	86470	1	2
214	2.39	sp|P05556|ITB1_HUMAN	3.759000078	798	88414.6	2	2
215	2.36	sp|P68366|TBA4A_HUMAN	15.85000008	448	49924	1	1
216	2.36	sp|Q14697|GANAB_HUMAN	3.390000015	944	106873.1	1	1
217	2.35	sp|P21266|GSTM3_HUMAN	5.333000049	225	26559.3	1	2
218	2.28	sp|P60866|RS20_HUMAN	15.12999982	119	13372.6	2	2
219	2.24	sp|P56537|IF6_HUMAN	5.714000016	245	26598.8	1	1
220	2.23	sp|P62424|RL7A_HUMAN	4.134999961	266	29995.4	1	3
221	2.21	sp|P43243|MATR3_HUMAN	3.187999874	847	94622.4	2	2
222	2.19	sp|O14684|PTGES_HUMAN	6.578999758	152	17102.1	1	1
223	2.17	sp|Q9H9B4|SFXN1_HUMAN	4.036999866	322	35619.1	1	1
224	2.17	sp|Q12965|MYO1E_HUMAN	1.805000007	1108	127061.1	1	1
225	2.15	sp|Q15029|U5S1_HUMAN	1.336999983	972	109434.8	1	2
226	2.14	sp|Q9BUJ2|HNRL1_HUMAN	1.75199993	856	95738	1	1
227	2.14	sp|Q16658|FSCN1_HUMAN	5.07100001	493	54529.5	2	2
228	2.12	sp|P08708|RS17_HUMAN	8.147999644	135	15550	1	1
229	2.1	sp|P23246|SFPQ_HUMAN	1.69699993	707	76149.1	1	2
230	2.1	sp|Q9UQE7|SMC3_HUMAN	1.150000002	1217	141540.7	1	1
231	2.09	sp|Q15365|PCBP1_HUMAN	17.1299994	356	37497.5	2	4
232	2.09	sp|P53992|SC24C_HUMAN	1.188000012	1094	118323.8	1	1
233	2.09	sp|O15173|PGRC2_HUMAN	8.519999683	223	23818.2	1	1
234	2.08	sp|O75533|SF3B1_HUMAN	1.150000002	1304	145829.1	1	1
235	2.08	sp|P15328|FOLR1_HUMAN	4.28000018	257	29818.9	1	1
236	2.07	sp|Q92598|HS105_HUMAN	3.497000039	858	96864.3	1	1
237	2.07	sp|P30050|RL12_HUMAN	9.091000259	165	17818.4	1	2
238	2.07	sp|O43175|SERA_HUMAN	2.813999914	533	56650	1	1
239	2.06	sp|Q99714|HCD2_HUMAN	6.51300028	261	26922.9	1	1
240	2.06	sp|P49792|RBP2_HUMAN	0.403199997	3224	358196.4	1	1
241	2.06	sp|P50416|CPT1A_HUMAN	1.81099996	773	88366.9	1	1
242	2.06	sp|P38117|ETFB_HUMAN	4.706000164	255	27843.4	1	1
243	2.05	sp|P15880|RS2_HUMAN	7.508999854	293	31324.2	2	2
244	2.05	sp|P62701|RS4X_HUMAN	3.421999887	263	29597.5	1	5
245	2.05	sp|P17812|PYRG1_HUMAN	2.030000091	591	66689.9	1	2
246	2.05	sp|Q9HCC0|MCCB_HUMAN	2.487000078	563	61332.7	1	2
247	2.04	sp|P05187|PPB1_HUMAN	3.364000097	535	57953.3	1	1
248	2.04	sp|Q13148|TADBP_HUMAN	4.348000139	414	44739.6	1	1
249	2.04	sp|P42765|THIM_HUMAN	7.052999735	397	41923.8	2	2
250	2.03	sp|P43246|MSH2_HUMAN	1.284999959	934	104742.3	1	1
252	2.02	sp|P52789|HXK2_HUMAN	1.527000032	917	102379.1	1	1
253	2.02	sp|Q9UHX1|PUF60_HUMAN	2.862000093	559	59875	1	5

No.	Unused	Acc	Coverage (%)	Length	Mass	#Unique Peptide	#Unique Spectrum
254	2.02	sp|Q9BM7|DHCR7_HUMAN	2.737000026	475	54489	1	1
255	2.02	sp|P48643|TCPE_HUMAN	2.218000032	541	59670.5	1	3
256	2.02	sp|P04040|CATA_HUMAN	2.466999926	527	59755.8	1	1
257	2.01	sp|Q9NSE4|SYIM_HUMAN	1.185999997	1012	113790.6	1	1
258	2.01	sp|P30040|ERP29_HUMAN	5.747000128	261	28993.2	1	1
259	2	sp|P63261|ACTG_HUMAN	41.33000076	375	41792.5	1	1
260	2	sp|P10412|H14_HUMAN	21.92000002	219	21865	1	2
261	2	sp|Q99729|ROAA_HUMAN	8.433999866	332	36224.8	2	1
262	2	sp|P12236|ADT3_HUMAN	6.711000204	298	32866	1	1
264	2	sp|Q13310|PABP4_HUMAN	1.707999967	644	70782.3	1	1
265	2	sp|P62263|RS14_HUMAN	8.608999848	151	16272.6	1	1
266	2	sp|P40925|MDHC_HUMAN	5.090000108	334	36425.8	1	1
267	2	sp|P33992|MCM5_HUMAN	1.771000028	734	82284.7	1	1
268	2	sp|P22234|PUR6_HUMAN	2.824000083	425	47078.8	1	3
269	2	sp|O43684|BUB3_HUMAN	4.267999902	328	37154.5	1	2
270	2	sp|O15533|TPSN_HUMAN	2.902000025	448	47625.3	1	1
271	2	sp|Q9NY93|DDX56_HUMAN	2.559000067	547	61588.9	1	1
273	2	sp|Q96RQ3|MCCA_HUMAN	1.516999956	725	80472.4	1	1
274	2	sp|Q96GQ7|DDX27_HUMAN	1.508000027	796	89834.5	1	1
275	2	sp|Q16891|MIC60_HUMAN	2.507000044	758	83677.1	1	1
276	2	sp|Q13409|DC1I2_HUMAN	3.291999921	638	71456.1	1	1
277	2	sp|Q01844|EWS_HUMAN	2.133999951	656	68478.2	1	2
278	2	sp|Q01813|PFKAP_HUMAN	2.422999963	784	85595.4	1	2
280	2	sp|P62318|SMD3_HUMAN	7.936999947	126	13916.2	1	1
281	2	sp|P61964|WDR5_HUMAN	4.191999882	334	36588.1	1	1
282	2	sp|P61221|ABCE1_HUMAN	2.002999932	599	67313.7	1	1
283	2	sp|P51991|ROA3_HUMAN	5.820000172	378	39595	1	1
284	2	sp|P46782|RS5_HUMAN	6.373000145	204	22876.2	1	2
285	2	sp|P46459|NSF_HUMAN	1.747000031	744	82593.6	1	1
286	2	sp|P16152|CBR1_HUMAN	5.776000023	277	30374.7	1	2
287	2	sp|P14314|GLU2B_HUMAN	1.893999986	528	59424.9	1	1
288	2	sp|O95336|6PGL_HUMAN	6.202000007	258	27546.5	1	1
289	2	sp|O76021|RL1D1_HUMAN	3.469000012	490	54972	1	1
290	2	sp|O75131|CPNE3_HUMAN	2.235000022	537	60130.2	1	1
291	2	sp|O60749|SNX2_HUMAN	3.08299996	519	58470.5	1	1
292	2	sp|O14828|SCAM3_HUMAN	4.611000046	347	38286.5	1	2
293	2	sp|O00303|EIF3F_HUMAN	4.761999846	357	37563.5	1	1
294	2	sp|Q9Y6E2|BZW2_HUMAN	2.147999965	419	48162	1	1
295	2	sp|Q9Y5M8|SRPRB_HUMAN	7.011000067	271	29701.9	1	1
296	2	sp|Q9Y4P3|TBL2_HUMAN	3.13199982	447	49797.4	1	1

No.	Unused	Acc	Coverage (%)	Length	Mass	#Unique Peptide	#Unique Spectrum
297	2	sp|Q9Y285|SYFA_HUMAN	2.755999938	508	57563.2	1	1
298	2	sp|Q9H936|GHC1_HUMAN	4.644000158	323	34469.8	1	1
299	2	sp|Q9BVC6|TM109_HUMAN	4.938000068	243	26209.6	1	3
301	2	sp|Q96HE7|ERO1A_HUMAN	2.991000004	468	54392.1	1	1
302	2	sp|Q969M3|YIPF5_HUMAN	4.668999836	257	27989	1	1
304	2	sp|Q8NC51|PAIRB_HUMAN	5.14700003	408	44965.2	1	3
306	2	sp|Q16563|SYPL1_HUMAN	4.247000068	259	28565	1	1
307	2	sp|Q15717|ELAV1_HUMAN	3.373999894	326	36091.6	1	1
308	2	sp|Q15041|AR6P1_HUMAN	4.926000163	203	23362.6	1	1
309	2	sp|Q14157|UBP2L_HUMAN	1.379999984	1087	114533.8	1	1
310	2	sp|Q14137|BOP1_HUMAN	1.876999997	746	83628.8	1	1
311	2	sp|Q13867|BLMH_HUMAN	4.396000132	455	52561.9	1	1
312	2	sp|Q01650|LAT1_HUMAN	3.550000116	507	55009.6	1	2
313	2	sp|P84098|RL19_HUMAN	8.673000336	196	23465.8	1	1
314	2	sp|P62913|RL11_HUMAN	7.864999771	178	20252.2	1	2
315	2	sp|P62910|RL32_HUMAN	9.629999846	135	15859.7	1	1
316	2	sp|P62861|RS30_HUMAN	16.94999933	59	6647.9	1	2
317	2	sp|P61353|RL27_HUMAN	6.617999822	136	15797.6	1	2
319	2	sp|P52565|GDIR1_HUMAN	7.353000343	204	23206.9	1	4
320	2	sp|P52209|6PGD_HUMAN	3.519999981	483	53139.6	1	1
321	2	sp|P47914|RL29_HUMAN	9.433999658	159	17751.9	1	2
322	2	sp|P46087|NOP2_HUMAN	1.724000089	812	89301.1	1	1
323	2	sp|P35268|RL22_HUMAN	10.15999988	128	14786.9	1	2
324	2	sp|P35080|PROF2_HUMAN	10.00000015	140	15046.2	1	1
325	2	sp|P29692|EF1D_HUMAN	8.540999889	281	31121.6	1	1
326	2	sp|P27338|AOFB_HUMAN	2.885000035	520	58762.5	1	1
327	2	sp|P25788|PSA3_HUMAN	4.706000164	255	28433	1	1
328	2	sp|P24534|EF1B_HUMAN	5.778000131	225	24763.5	1	2
329	2	sp|P12268|IMDH2_HUMAN	2.528999932	514	55804.5	1	2
330	2	sp|P11413|G6PD_HUMAN	3.106999956	515	59256.3	1	1
331	2	sp|P11166|GTR1_HUMAN	2.033000067	492	54083.3	1	3
332	2	sp|P05198|IF2A_HUMAN	3.810000047	315	36111.8	1	1
333	2	sp|P05165|PCCA_HUMAN	2.06000004	728	80058.3	1	1
334	2	sp|O75489|NDUS3_HUMAN	4.924000055	264	30241.2	1	1
335	2	sp|O75396|SC22B_HUMAN	6.511999667	215	24593.1	1	2
336	2	sp|O60568|PLOD3_HUMAN	1.896999963	738	84784.5	1	1
337	2	sp|O14980|XPO1_HUMAN	1.11999996	1071	123385	1	1
338	2	sp|O00567|NOP56_HUMAN	2.188999951	594	66049.3	1	1
339	1.92	sp|P49748|ACADV_HUMAN	1.83199998	655	70389.6	1	1
340	1.89	sp|Q15125|EBP_HUMAN	4.348000139	230	26352.6	1	1

No.	Unused	Acc	Coverage (%)	Length	Mass	#Unique Peptide	#Unique Spectrum
341	1.89	sp|P78417|GSTO1_HUMAN	5.809000134	241	27565.6	1	1
342	1.89	sp|Q9NZT2|OGFR_HUMAN	1.77299995	677	73324	1	1
344	1.87	sp|P24752|THIL_HUMAN	3.044000082	427	45199.2	1	2
345	1.85	sp|P42766|RL35_HUMAN	11.37999967	123	14551.4	1	1
346	1.84	sp|P20700|LMNB1_HUMAN	1.876999997	586	66407.7	1	1
347	1.82	sp|P63244|RACK1_HUMAN	2.524000034	317	35076.5	1	1
348	1.8	sp|Q02543|RL18A_HUMAN	7.385999709	176	20762.2	1	2
349	1.8	sp|Q99536|VAT1_HUMAN	2.799000032	393	41920	1	1
350	1.79	sp|Q9UGI8|TES_HUMAN	4.512999952	421	47996.1	1	1
351	1.78	sp|P04899|GNAI2_HUMAN	3.099000081	355	40450.5	1	2
352	1.77	sp|P45974|UBP5_HUMAN	1.748000085	858	95785.4	1	2
353	1.76	sp|Q9NRG9|AAAS_HUMAN	2.747000009	546	59573.6	1	1
354	1.72	sp|P08574|CY1_HUMAN	4.922999814	325	35421.6	1	3
355	1.7	sp|Q9NYH9|UTP6_HUMAN	2.512999997	597	70193.2	1	1
356	1.66	sp|Q12792|TWF1_HUMAN	3.714000061	350	40282.4	1	1
357	1.61	sp|Q9Y265|RUVB1_HUMAN	3.07	456	50227.6	1	1
358	1.6	sp|P61619|S61A1_HUMAN	2.311000042	476	52264.2	1	1
359	1.59	sp|Q13435|SF3B2_HUMAN	1.67599991	895	100226.9	1	1
360	1.59	sp|P46777|RL5_HUMAN	4.713999853	297	34362.4	1	1
361	1.58	sp|O60664|PLIN3_HUMAN	4.146999866	434	47074.7	1	1
362	1.55	sp|Q9Y3I0|RTCB_HUMAN	2.177999914	505	55209.9	1	1
363	1.54	sp|P15311|EZRI_HUMAN	5.973000079	586	69412.3	1	1
364	1.5	sp|P49756|RBM25_HUMAN	2.135000005	843	100184.5	1	1
365	1.49	sp|P13798|ACPH_HUMAN	1.775999926	732	81223.9	1	2
366	1.47	sp|O95816|BAG2_HUMAN	5.21299988	211	23771.7	1	1
367	1.47	sp|Q3LXA3|TKFC_HUMAN	3.826000169	575	58946.5	1	1
369	1.43	sp|P33993|MCM7_HUMAN	1.807999983	719	81307.2	1	1
370	1.43	sp|Q96CX2|KCD12_HUMAN	4.922999814	325	35700.4	1	1
371	1.4	sp|Q13162|PRDX4_HUMAN	8.487000316	271	30539.6	1	1
372	1.38	sp|P10909|CLUS_HUMAN	3.562999889	449	52494.2	1	1
373	1.37	sp|P30740|ILEU_HUMAN	2.902000025	379	42741.4	1	1
374	1.36	sp|P48507|GSH0_HUMAN	4.744999856	274	30726.7	1	1
375	1.35	sp|Q9BK6|TMED9_HUMAN	3.830000013	235	27277.2	1	1
377	1.33	sp|P21980|TGM2_HUMAN	1.892000064	687	77328.2	1	1
378	1.32	sp|P00390|GSHR_HUMAN	2.29899995	522	56256.6	1	1
379	1.32	sp|Q9UNE2|RPH3L_HUMAN	3.810000047	315	34463.7	1	1
380	1.31	sp|Q02790|FKBP4_HUMAN	1.961000077	459	51804.2	1	1
381	1.3	sp|P15559|NQO1_HUMAN	4.744999856	274	30867.4	1	1
